# Supplementary material for: Inhibitory medial zona incerta pathway drives exploratory behavior by inhibiting glutamatergic cuneiform neurons
Source: Nat Commun. 2024 Feb 7;15:1160. doi: 10.1038/s41467-024-45288-x (PMC10850156; doi:10.1038/s41467-024-45288-x)
Supplement: Supplementary file 1 — Supplementary Information [file 41467_2024_45288_MOESM1_ESM.pdf]

Supplementary information for

Inhibitory medial zona incerta pathway drives exploratory behavior by inhibiting glutamatergic cuneiform neurons.

AUTHORS

Sandeep Sharma[1,2], Cecilia A. Badenhorst[1,2], Donovan M. Ashby[1,3], Stephanie A. Di Vito[1], Michelle A. Tran[1,2], Zahra Ghavasieh[1], Gurleen K. Grewal[1], Cole R. Belway[1], Alexander McGirr[1,3], Patrick J. Whelan[1,2*]

AFFILIATIONS

[1]Hotchkiss Brain Institute, University of Calgary, Calgary, AB, Canada, T2N 4N1 [2]Faculty of Veterinary Medicine, University of Calgary, Calgary, AB, Canada, T2N 4N1 [3]Department of Psychiatry, Cumming School of Medicine, University of Calgary, Calgary, AB, Canada, T2N 4N1

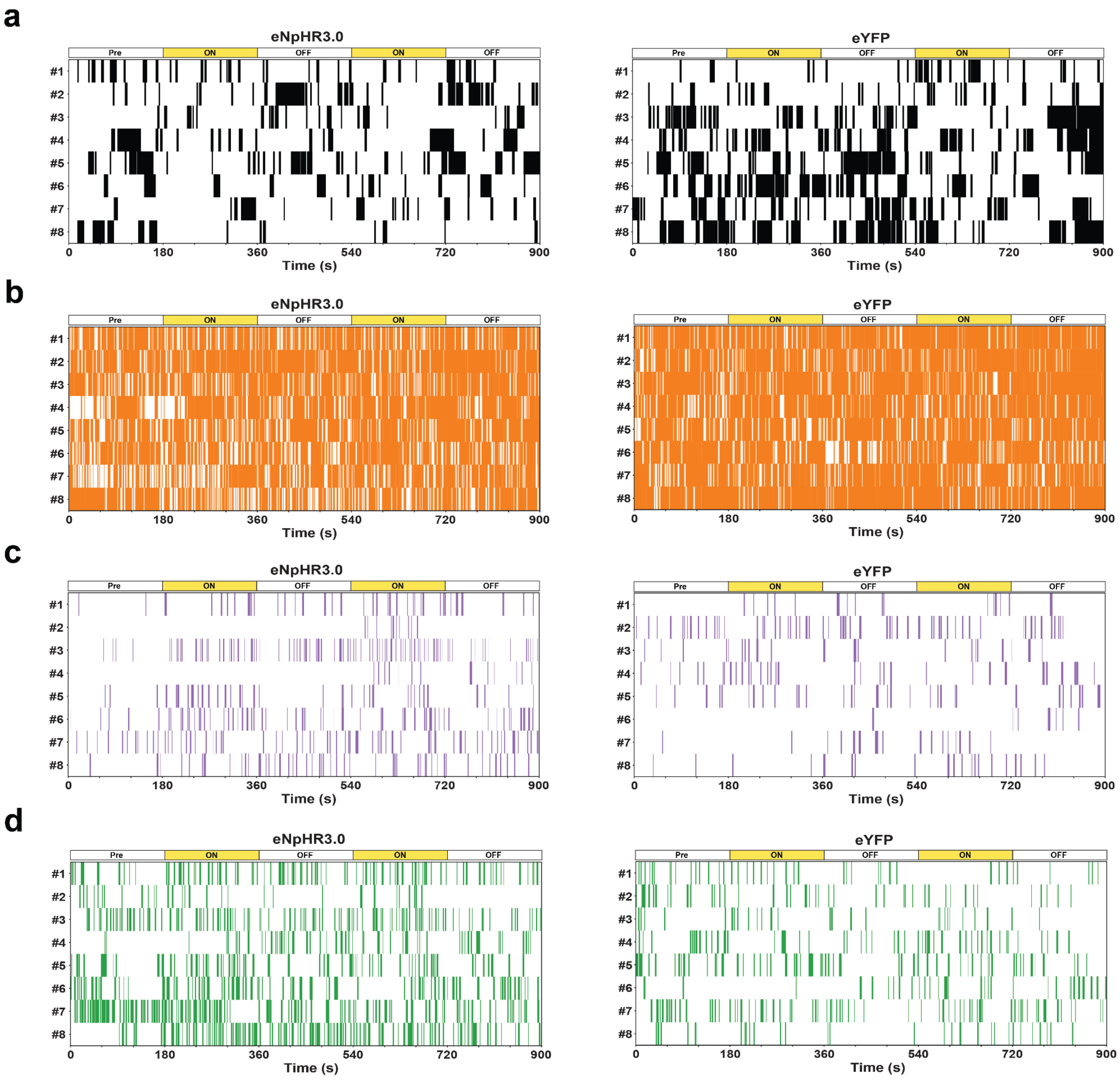

Supplementary Figure 1: Ethograms showing no significant change in behavior for grooming, surveying, rearing, and locomotion following photoinhibition of the CnF vGLUT2 neurons. Ethograms showing **a,** grooming (black), **b,** surveying (orange), **c,** rearing (purple) and **d,** locomotion (green) events before (Pre), during (ON), and after (OFF) yellow laser light (561 nm) inhibition of vGlut2 neurons in the CnF. A total of two trials of photoinhibition were conducted in a single session per mouse. Number of animals for the ethogram (eNpHR3.0: *n* = 8 mice; eYFP: *n* = 8 mice).

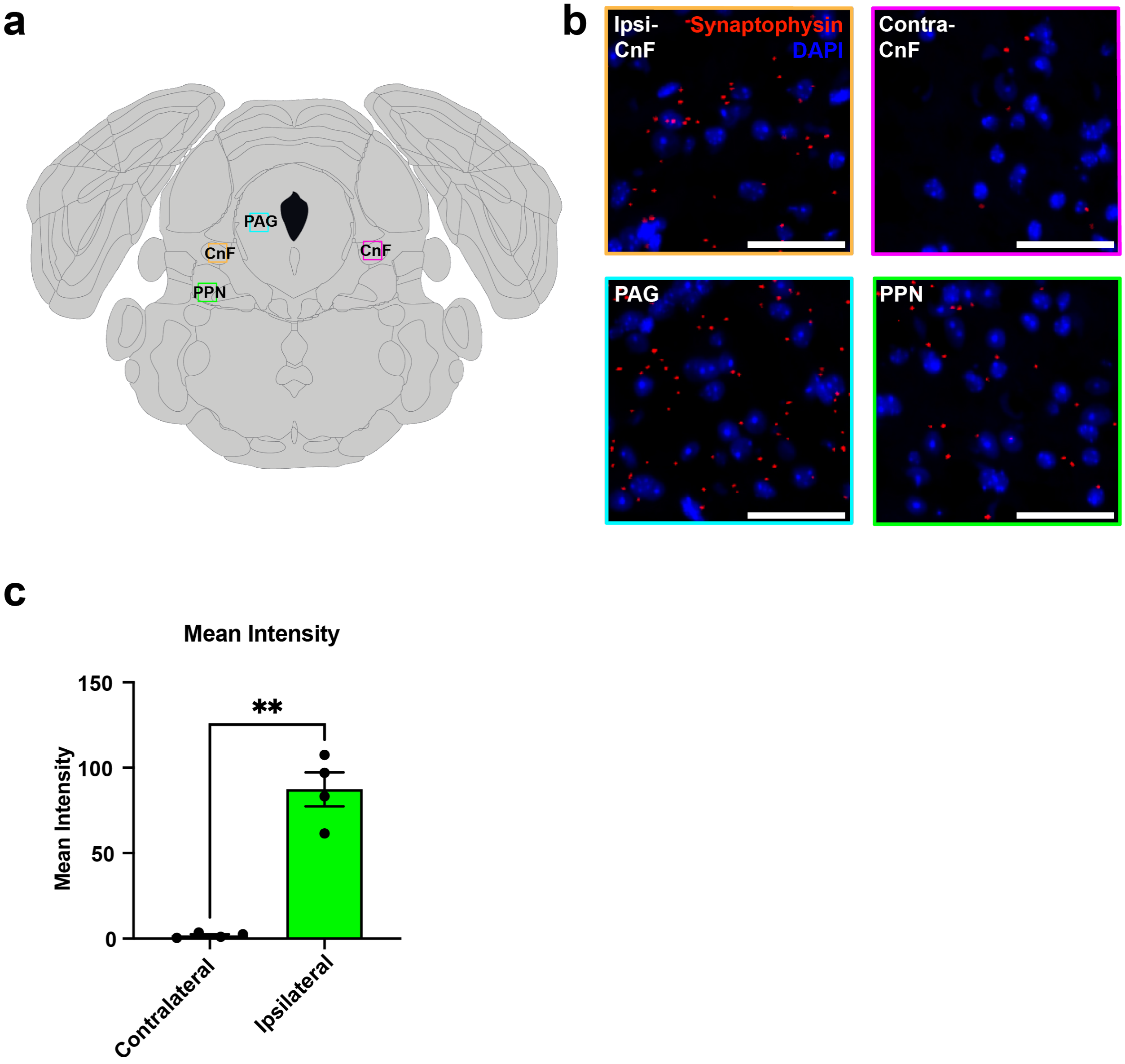

Supplementary Figure 2: Anatomical tracing of mZI GABAergic projections. a, Atlas overlay showing anatomical regions of interest: CnF (ipsilateral in orange and contralateral in magenta), PPN (ipsilateral in green) and PAG (ipsilateral in aqua blue). **b,** The top panel of images shows insets of ipsilateral CnF receiving GABAergic synaptic puncta (DAPI in blue and synaptophysin in red) and contralateral CnF with a sparse presence of synaptic puncta. The bottom panels depict inserts from the ipsilateral PAG and PPN, with synaptic puncta. **c**, The mZI GABAergic projections to the CnF are dominantly ipsilateral, with significantly higher mean fluorescence intensity (n = 5 mice, $p = 0.0001$, two-sided Unpaired t-test). Data are presented as mean +/- SEM. Scale bars are 50 µm unless otherwise indicated. Atlas image adapted from the Allen Mouse Brain Atlas[1,2]. Mouse brain illustration created with BioRender.com.

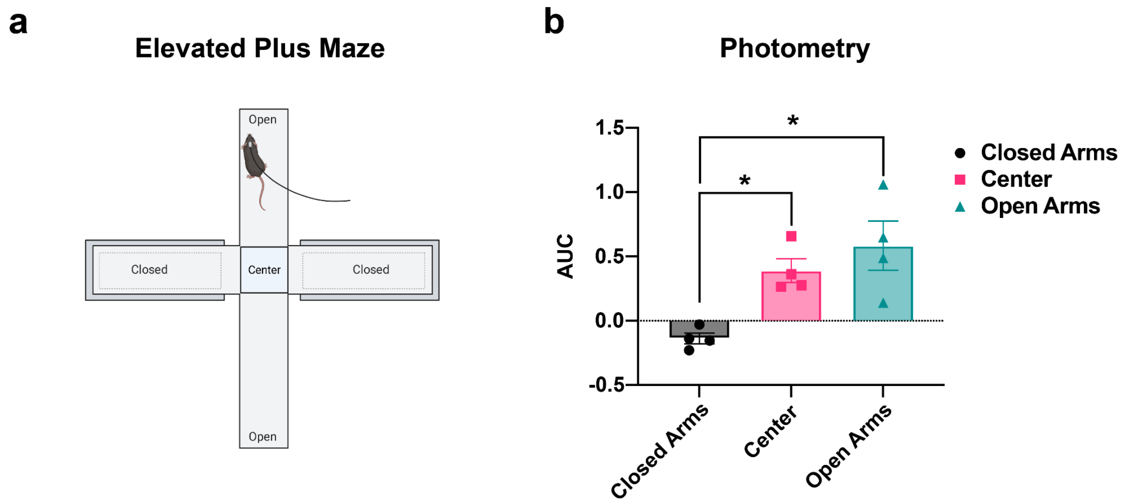

Supplementary Figure 3: mZI inhibitory neurons are active during exploratory behavior in the EPM. **a**, Schematic for the elevated plus maze used during fiber photometry. **b**, Area under the curve (AUC) showing increased calcium transients during exploration of the open arms and center (*n* = 4 mice, $F_{(2,6)}$ = 9.84, *p* = 0.013, one-way repeated measures ANOVA). *Post hoc* analysis shows a significant difference between the AUC of the center (*M* = 0.39 ± 0.092) and closed arms (*M* = -0.14 ± 0.041, *p* = 0.046, Tukey's multiple comparison test) and between the closed and the open arms (*M* = 0.58 ± 0.19, *p* = 0.012, Tukey's multiple comparison test). Data are presented as mean +/- SEM. Elevated plus maze diagram created with BioRender.com. *$p < 0.05$

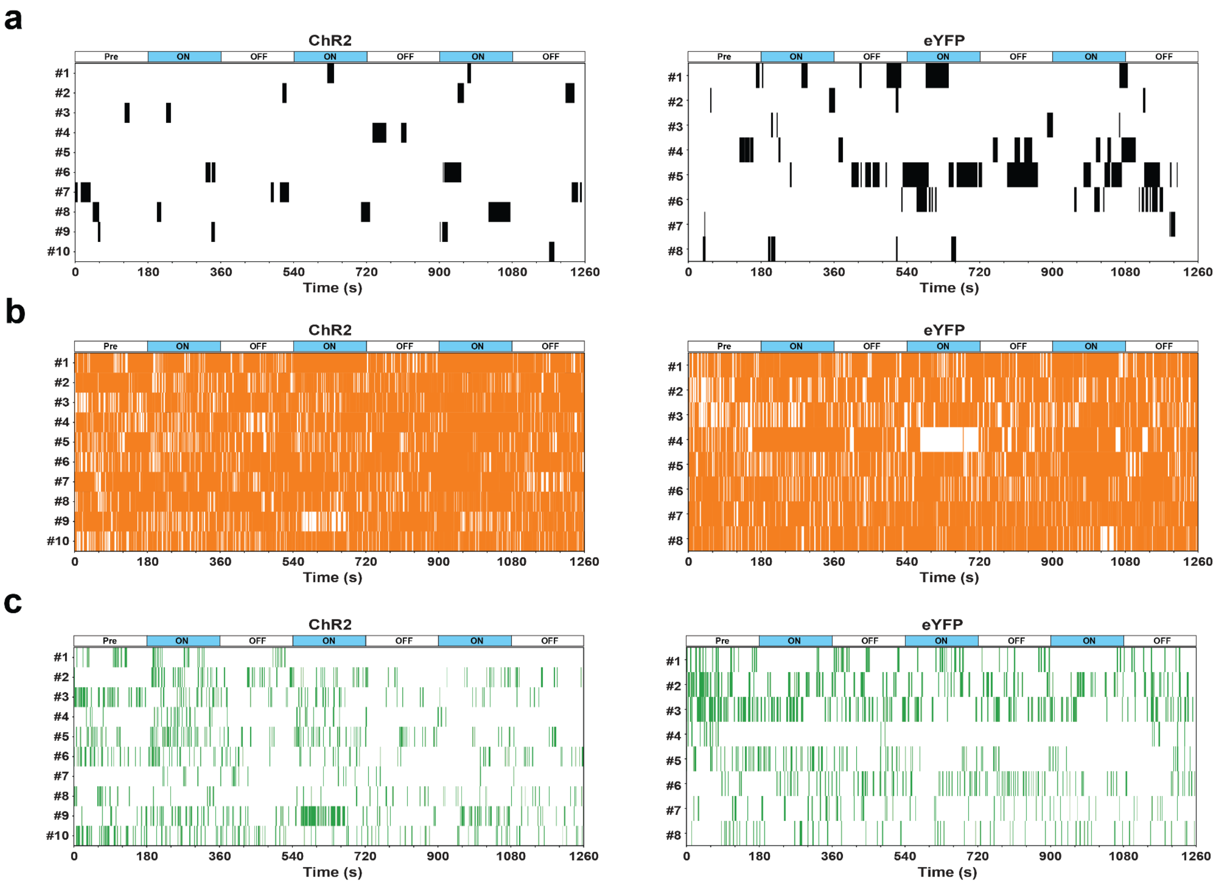

Supplementary Figure 4. Photostimulation of mZI GABAergic terminals in the CnF showed no change in the ethograms for the grooming, surveying and locomotion behaviors. Ethograms showing **a,** grooming (black), **b,** surveying (orange), and **c,** locomotion (green) events before (Pre), during (ON), and after (OFF) blue laser light (473 nm) stimulation of the CnF.A total of three trials of photostimulation were conducted in a single session per mouse. Number of animals for the ethogram (ChR2: *n* = 10 mice; eYFP: *n* = 8 mice).

Mice Strain	Full Name	Source		
vGLUT2-IRES-Cre	B6J.129S6(FVB)-$\mathit{Slc17a6}^{tm2(cre)Lowl}$/ MwarJ	The Jackson Laboratory (#028863)		
vGAT-IRES-Cre	B6J.129S6(FVB)-Slc32a1$^{tm2(cre)Lowl}$/ MwarJ	The Jackson Laboratory (#028862)		
**Virus**	**Full Name**	**Source**	**Lot Number**	**Titer (GC/mL)**
AAV-DIO-eNpHR3.0-eYFP	AAV9-EF1a-DIO-eNpHR3.0-eYFP	Addgene (#26966-AAV9)	v44904	2.2×10^{13}
AAV-DIO-eYFP	AAV9-Ef1a-DIO-eYFP	Addgene (#27056-AAV9)	v44841	2.2×10^{13}
AAV-DIO-ChR2-eYFP	AAV9-EF1a-DIO-hChR2(H134R)-EYFP-WPRE-HGHpA	Addgene (#20298-AAV9)	v45835	1.8×10^{13}
AAV-CAG.Flex.GCaMP6s	AAV9-CAG.Flex.GCaMP6s	Penn Vector Core	CS1257	4.6×10^{13}
AAV-Flex-mGFP-2A-Synaptophysin-mRuby	AAV-DJ-hSyn-Flex-mGFP-2A-Synaptophysin-mRUBY	Stanford Gene Vector and Virus Core (#GVVC-AAV-100)	5657	4.7×10^{12}
AAVrg-DIO-eYFP	AAVrg-Ef1a-DIO-eYFP	Addgene (#27056-AAVrg)	v120252	2.4×10^{13}
AAVrg-DIO-ChR2-eYFP	AAVrg-EF1a-DIO-hChR2(H134R)-EYFP-WPRE-HGHpA	Addgene (#20298-AAVrg)	v32674	1.0×10^{13}
**Antibody**		**Source**	**Lot Number**	**Dilution**
Chicken anti-Green Fluorescent Protein		Aves labs (#GFP-1010)	GFP3717982	1:3000
Goat anti-Choline Acetyltransferase		Millipore Sigma (#AB144P)	3789368-A	1:500
Alexa Fluor® 488 AffiniPure Donkey Anti-Chicken IgY (IgG) (H+L)		Jackson ImmunoResearch (#703-545-155)	154923	1:1000
Alexa Fluor® 647 Donkey anti-Goat IgG (H+L) Cross-Adsorbed Secondary Antibody		ThermoFisher Scientific (#A-21447)	2273780	1:1000

Supplementary Table 1: Table of mice, viruses and antibodies used in the experiments.

REFERENCES

1. Lein, E. S. *et al.* Genome-wide atlas of gene expression in the adult mouse brain. *Nature* **445**, 168–176 (2007).
2. Goldowitz, D. Allen Reference Atlas. A Digital Color Brain Atlas of the C57BL/6J Male Mouse - by H. W. Dong. *Genes, Brain and Behavior* vol. 9 128–128 Preprint at https://doi.org/10.1111/j.1601-183x.2009.00552.x (2010).
