# Supplementary material for: Inhibitory medial zona incerta pathway drives exploratory behavior by inhibiting glutamatergic cuneiform neurons
Source: Nat Commun. 2024 Feb 7;15:1160. doi: 10.1038/s41467-024-45288-x (PMC10850156; doi:10.1038/s41467-024-45288-x)
Supplement: Supplementary file 3 — Description of Additional Supplementary Files [file 41467_2024_45288_MOESM3_ESM.pdf]

Description of Additional Supplementary Files

File Name: Supplementary Software
Description: The scripts used for this manuscript and divided into Behavior, Photometry, and Microscopy folders containing documentation, scripts, and examples.
