# Supplementary figures and images for: Inhibitory medial zona incerta pathway drives exploratory behavior by inhibiting glutamatergic cuneiform neurons
Source: Nat Commun. 2024 Feb 7;15:1160. doi: 10.1038/s41467-024-45288-x (PMC10850156; doi:10.1038/s41467-024-45288-x)

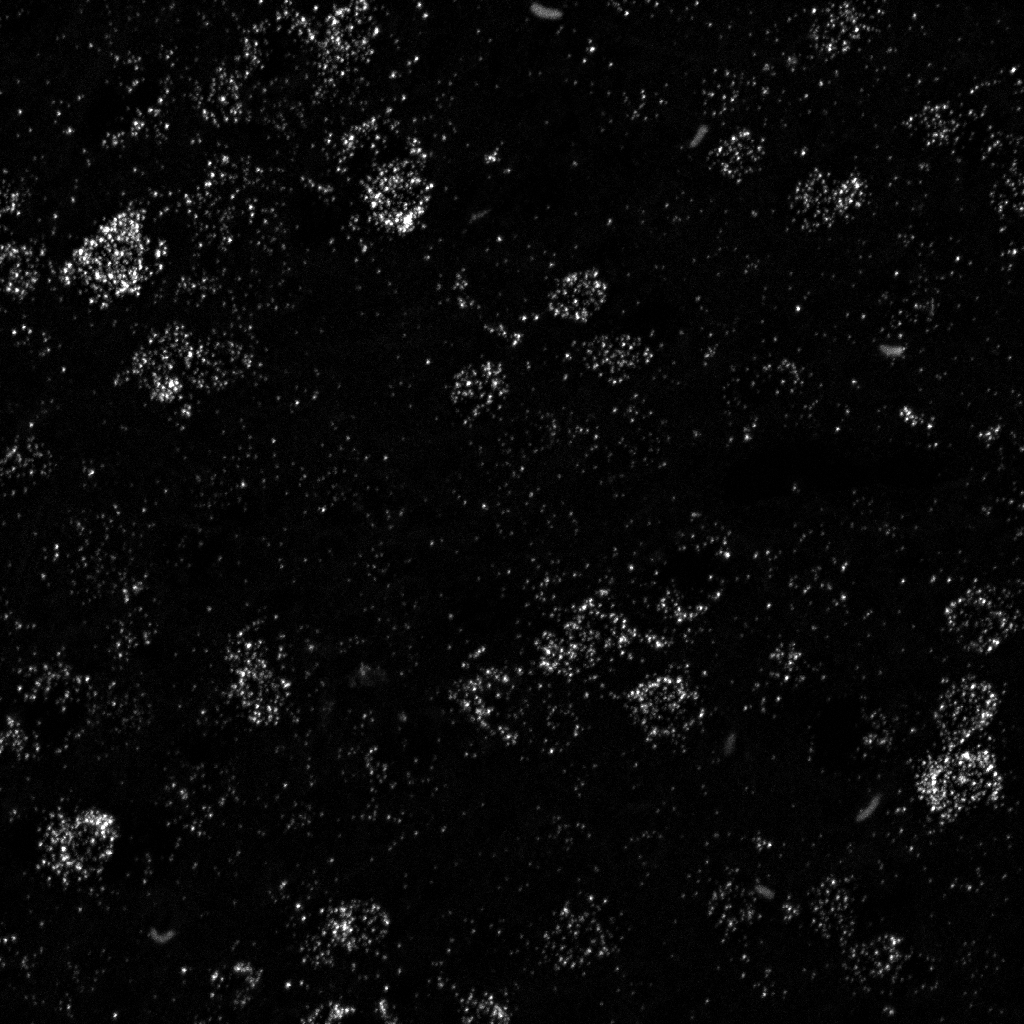

Supplement: Supplementary file 4 — Supplementary Software [file 41467_2024_45288_MOESM4_ESM.zip › Microscope scripts/sample/Qupath input/210521_0811_1_1_slide1pt4_vglut2_018_Merged_final.tif]

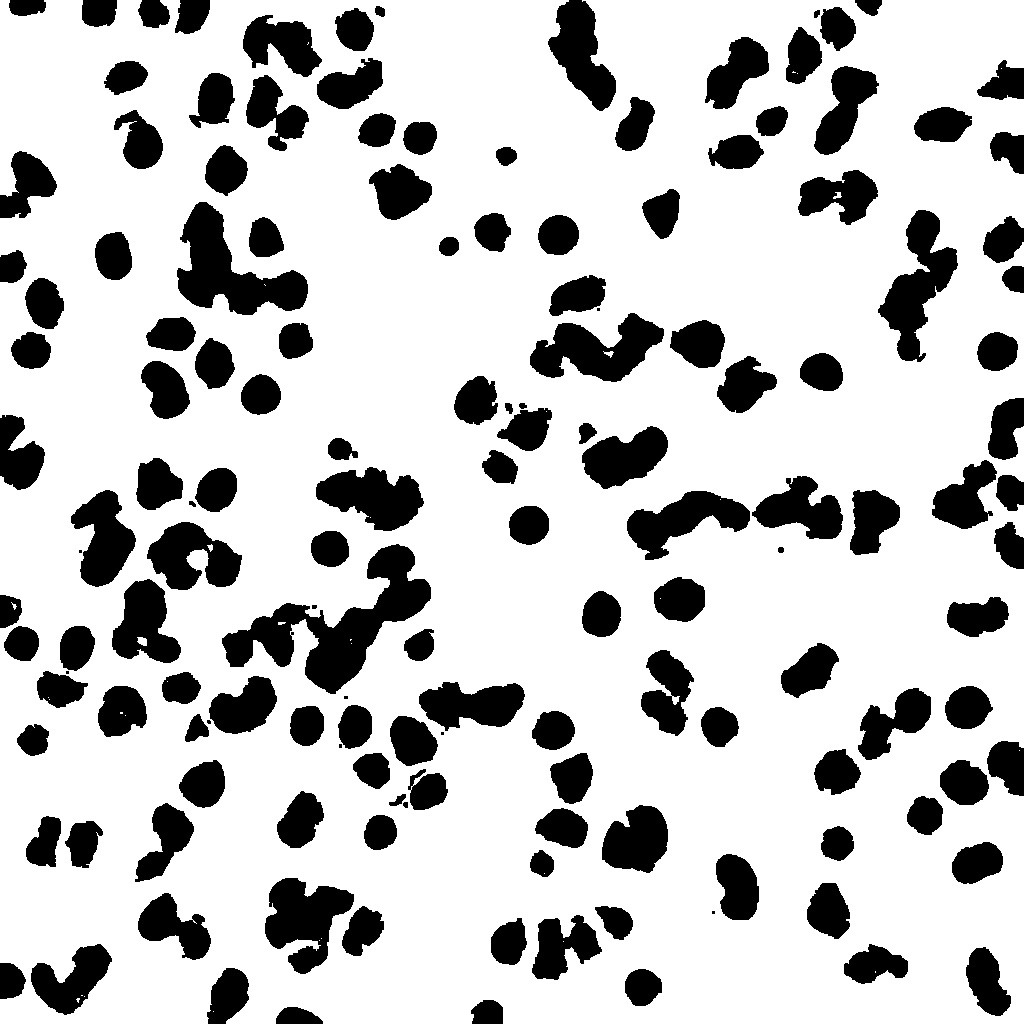

Supplement: Supplementary file 4 — Supplementary Software [file 41467_2024_45288_MOESM4_ESM.zip › Microscope scripts/sample/hdf2tif/210521_0811_1_1_slide1pt4_vglut2_018_Merged.tif]

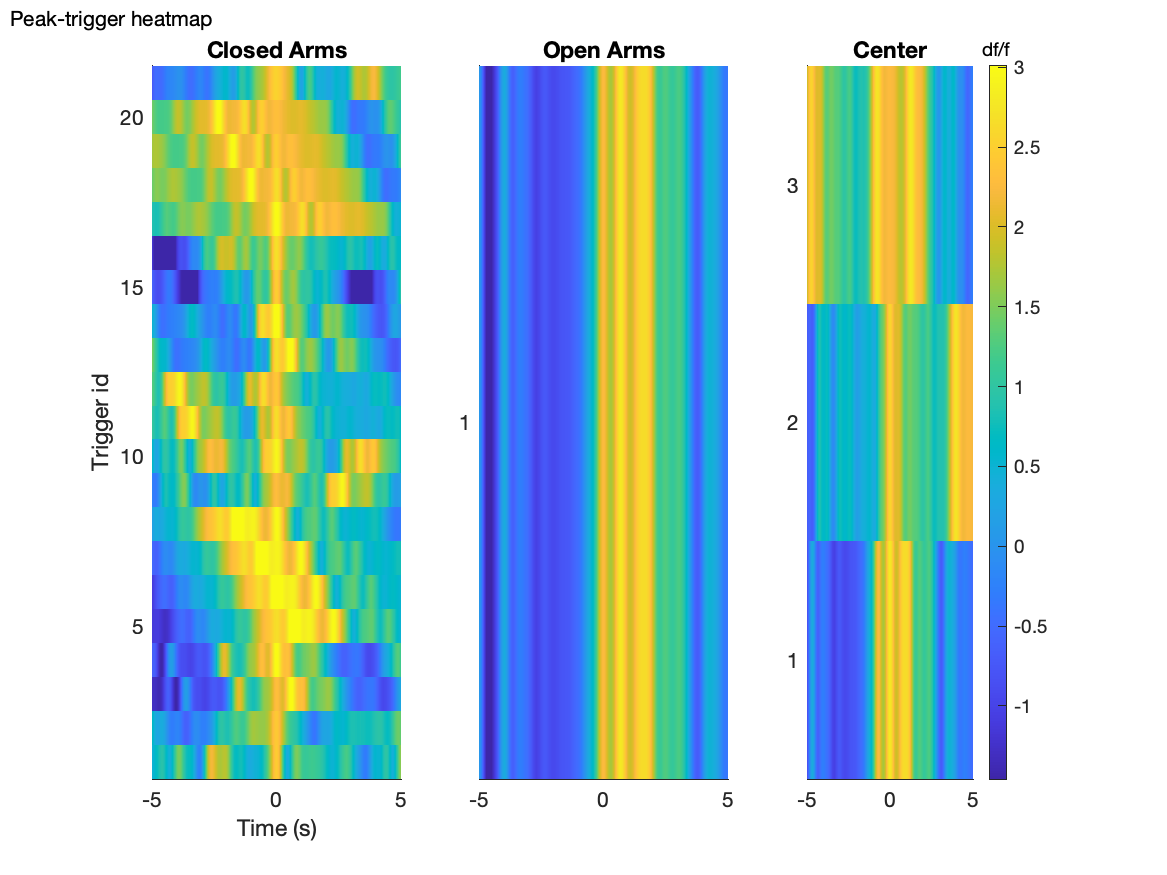

Supplement: Supplementary file 4 — Supplementary Software [file 41467_2024_45288_MOESM4_ESM.zip › Fiber Photometry Software/Outputs - from Sample Data/EPM outputs/Peaktrigger heatmap.png]

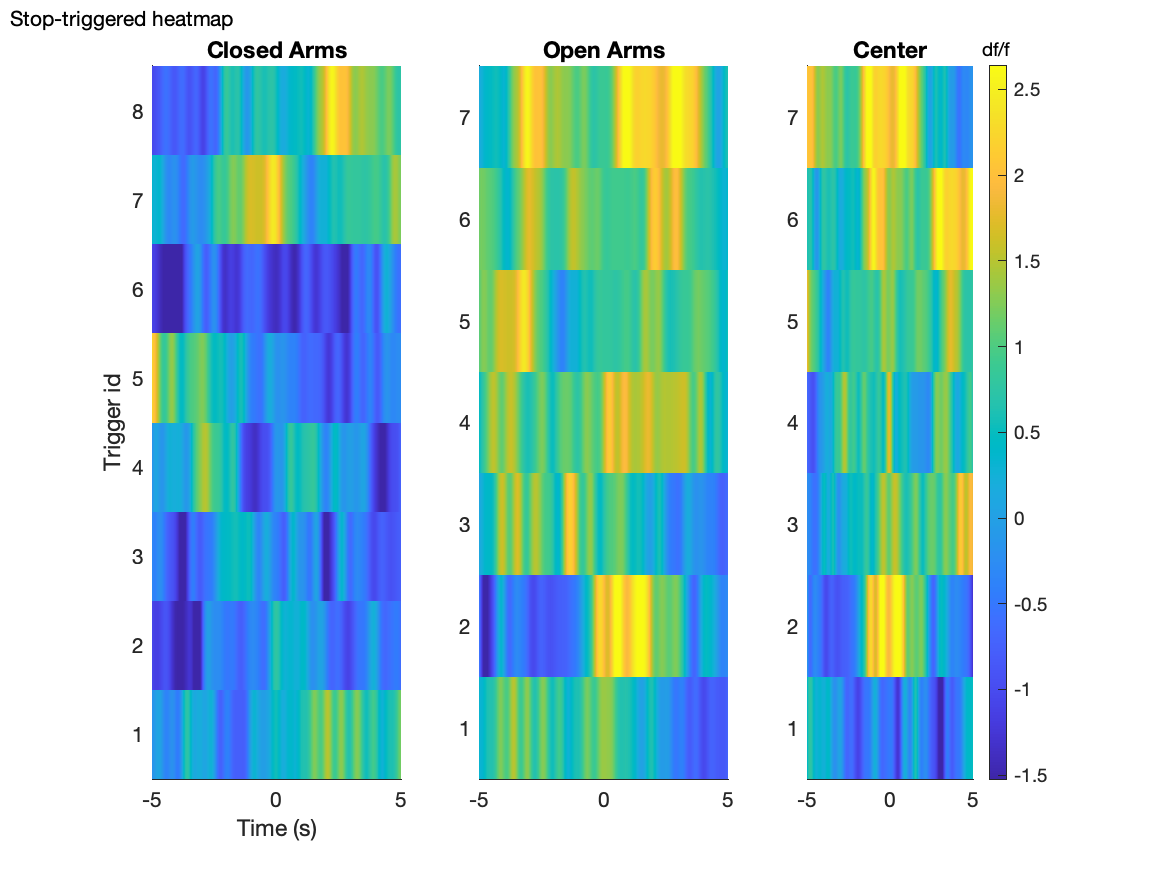

Supplement: Supplementary file 4 — Supplementary Software [file 41467_2024_45288_MOESM4_ESM.zip › Fiber Photometry Software/Outputs - from Sample Data/EPM outputs/Stoptriggered heatmap.png]

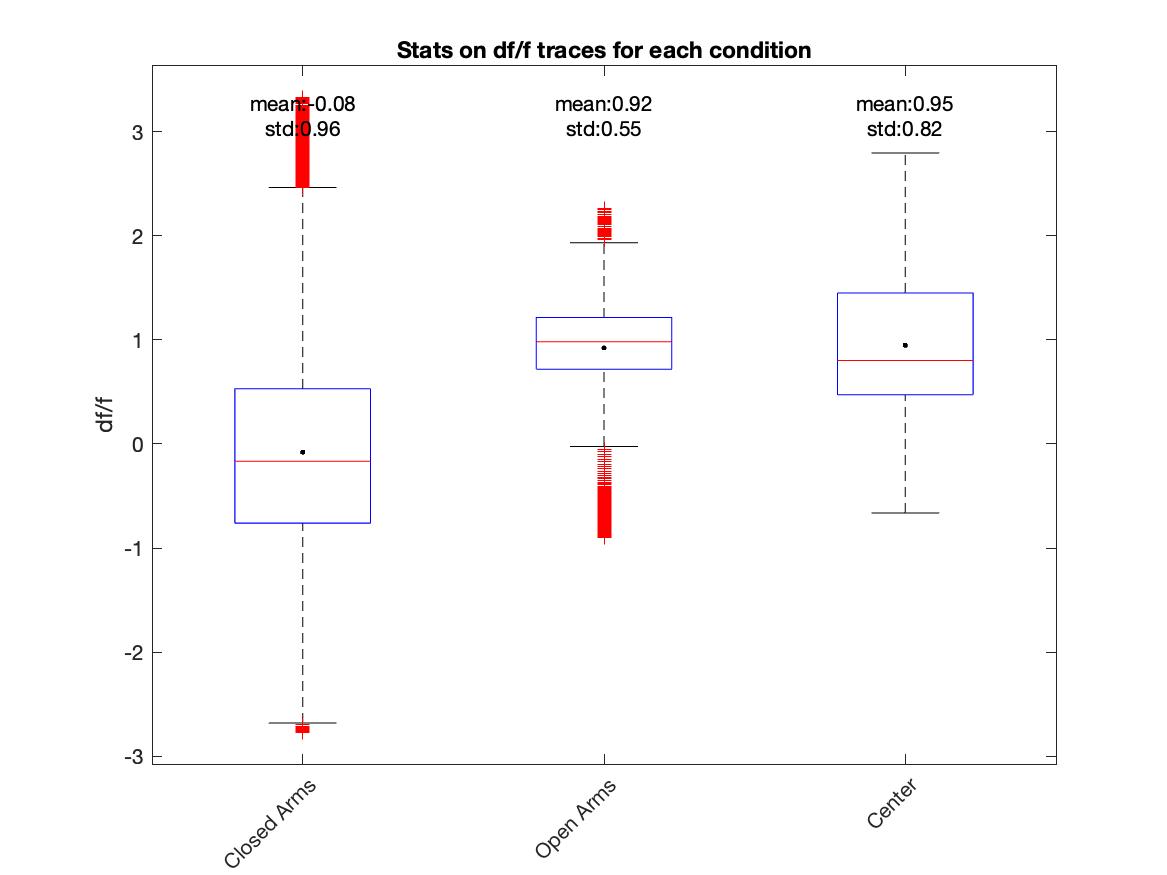

Supplement: Supplementary file 4 — Supplementary Software [file 41467_2024_45288_MOESM4_ESM.zip › Fiber Photometry Software/Outputs - from Sample Data/EPM outputs/FPA Boxplot.png]

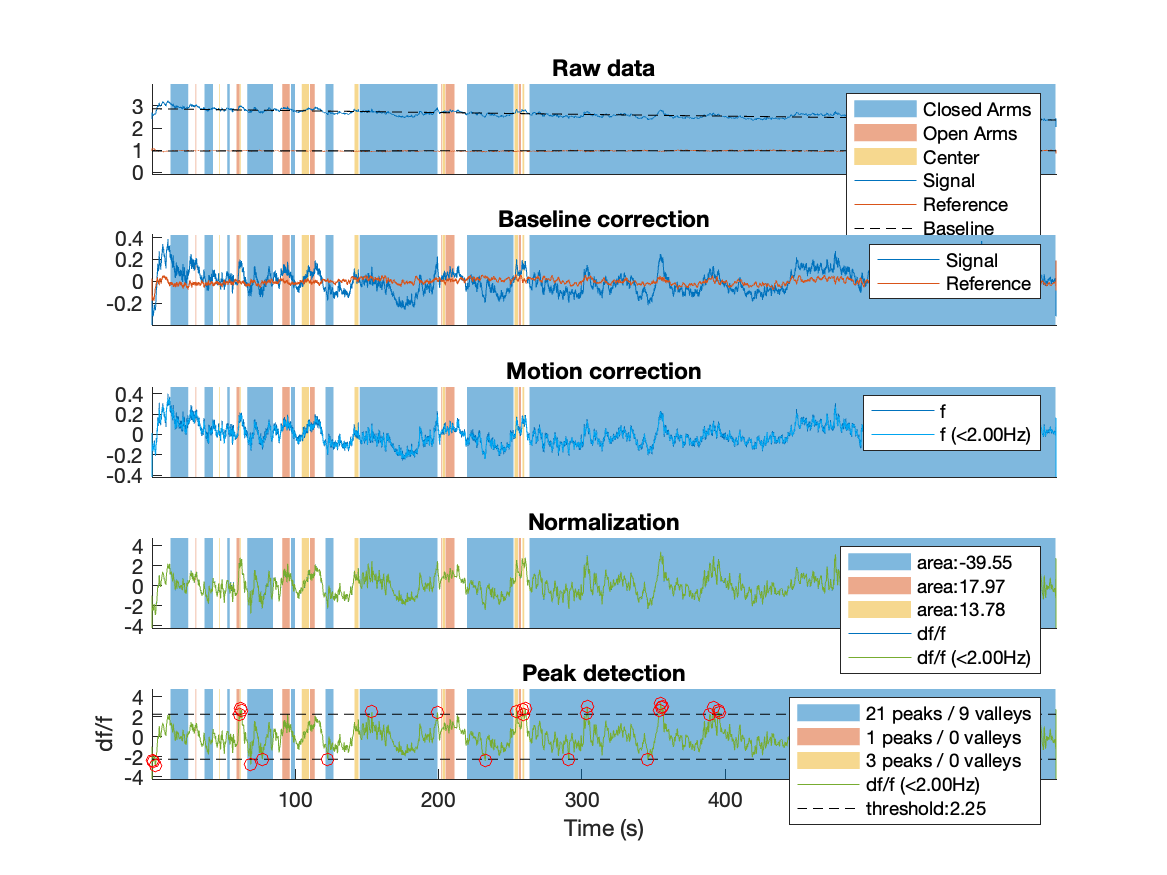

Supplement: Supplementary file 4 — Supplementary Software [file 41467_2024_45288_MOESM4_ESM.zip › Fiber Photometry Software/Outputs - from Sample Data/EPM outputs/FPA dff.png]

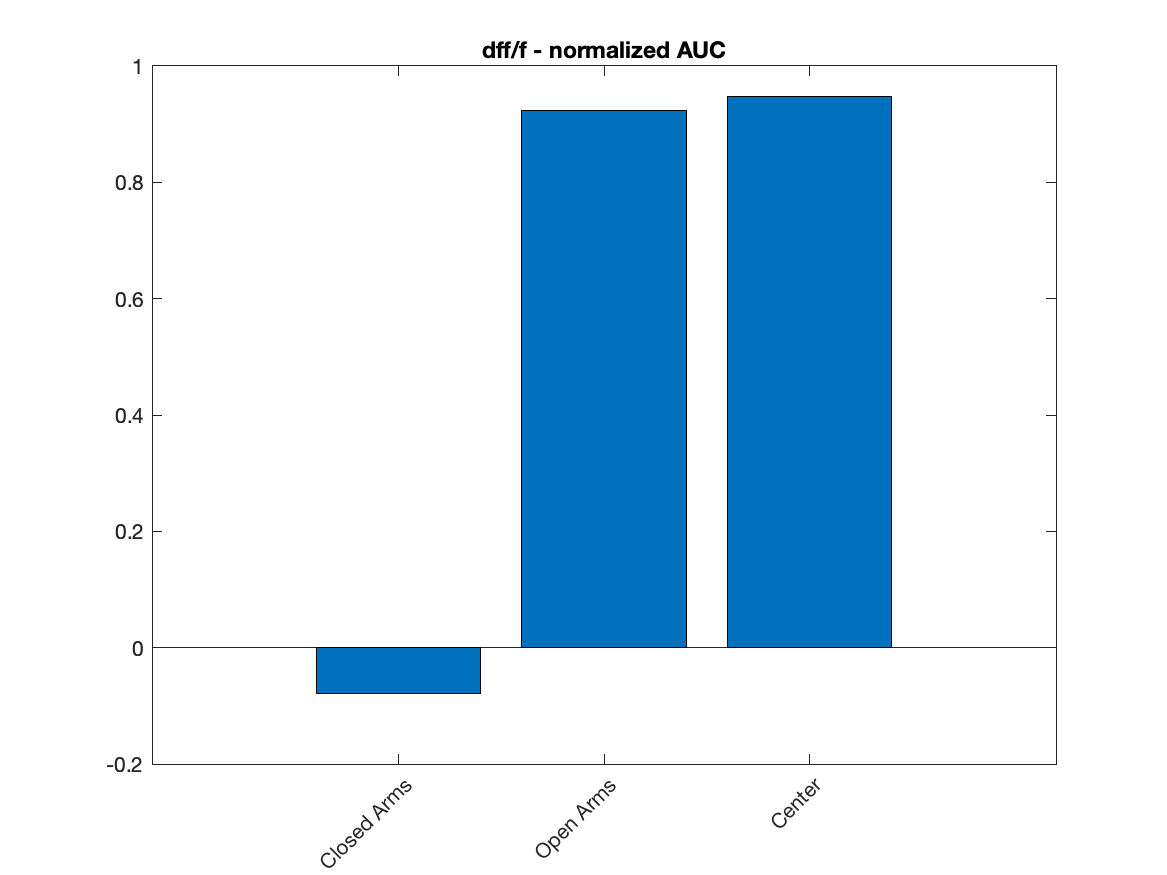

Supplement: Supplementary file 4 — Supplementary Software [file 41467_2024_45288_MOESM4_ESM.zip › Fiber Photometry Software/Outputs - from Sample Data/EPM outputs/FPA Normalized area under the curve.png]

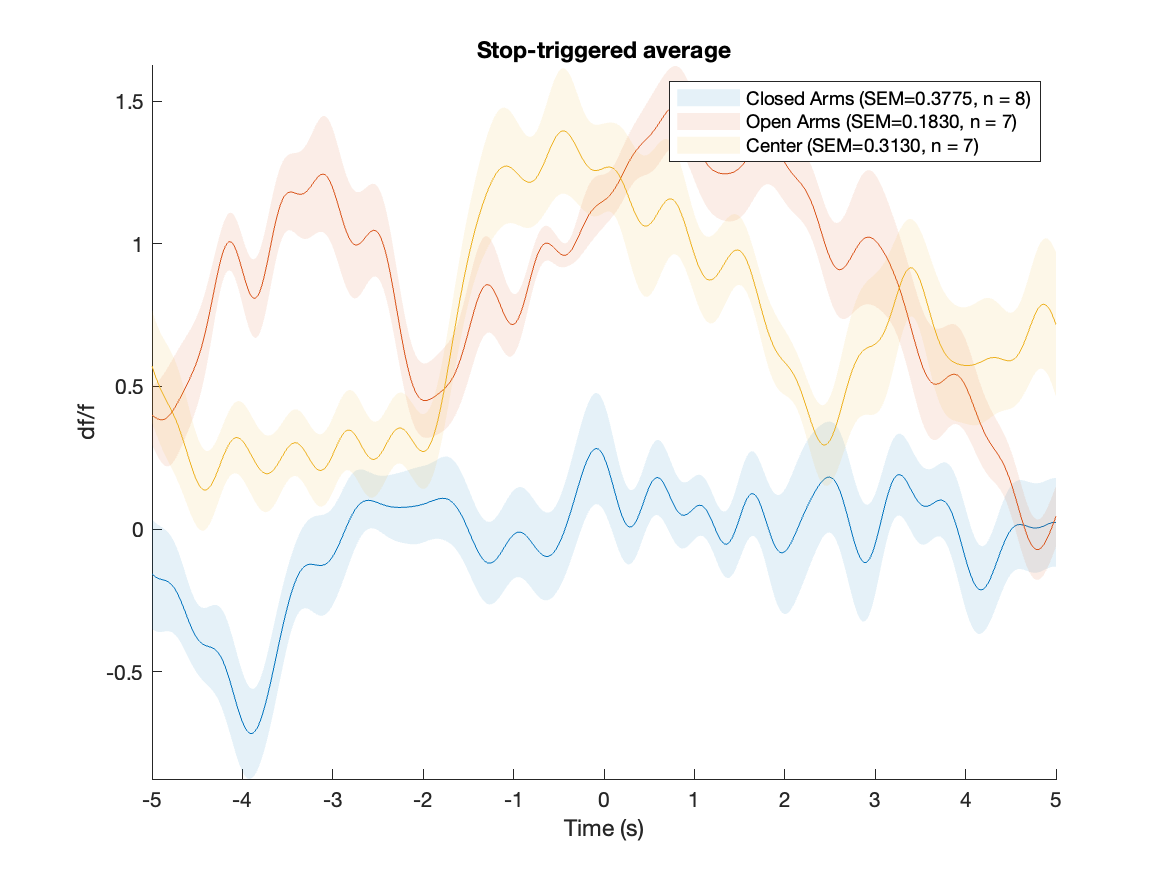

Supplement: Supplementary file 4 — Supplementary Software [file 41467_2024_45288_MOESM4_ESM.zip › Fiber Photometry Software/Outputs - from Sample Data/EPM outputs/FPA stoptriggered average.png]

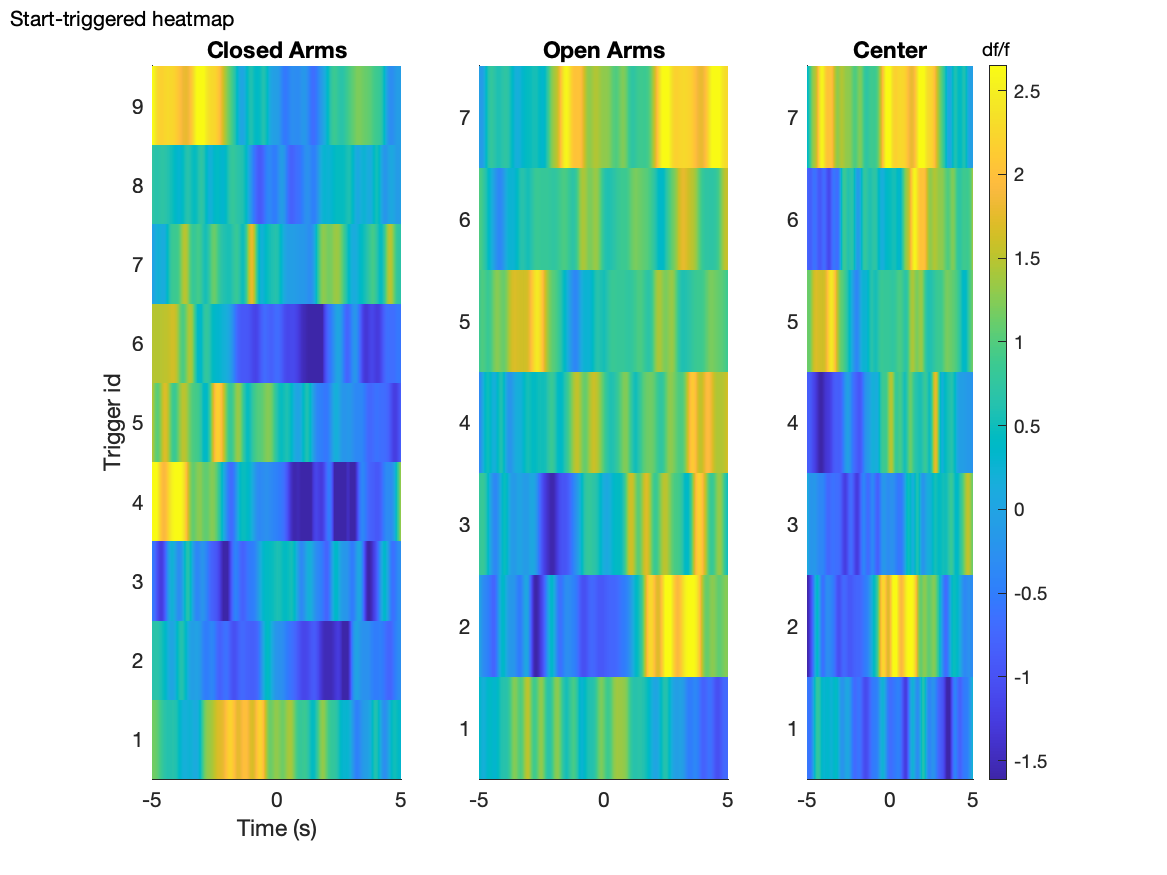

Supplement: Supplementary file 4 — Supplementary Software [file 41467_2024_45288_MOESM4_ESM.zip › Fiber Photometry Software/Outputs - from Sample Data/EPM outputs/Starttriggered heatmap.png]

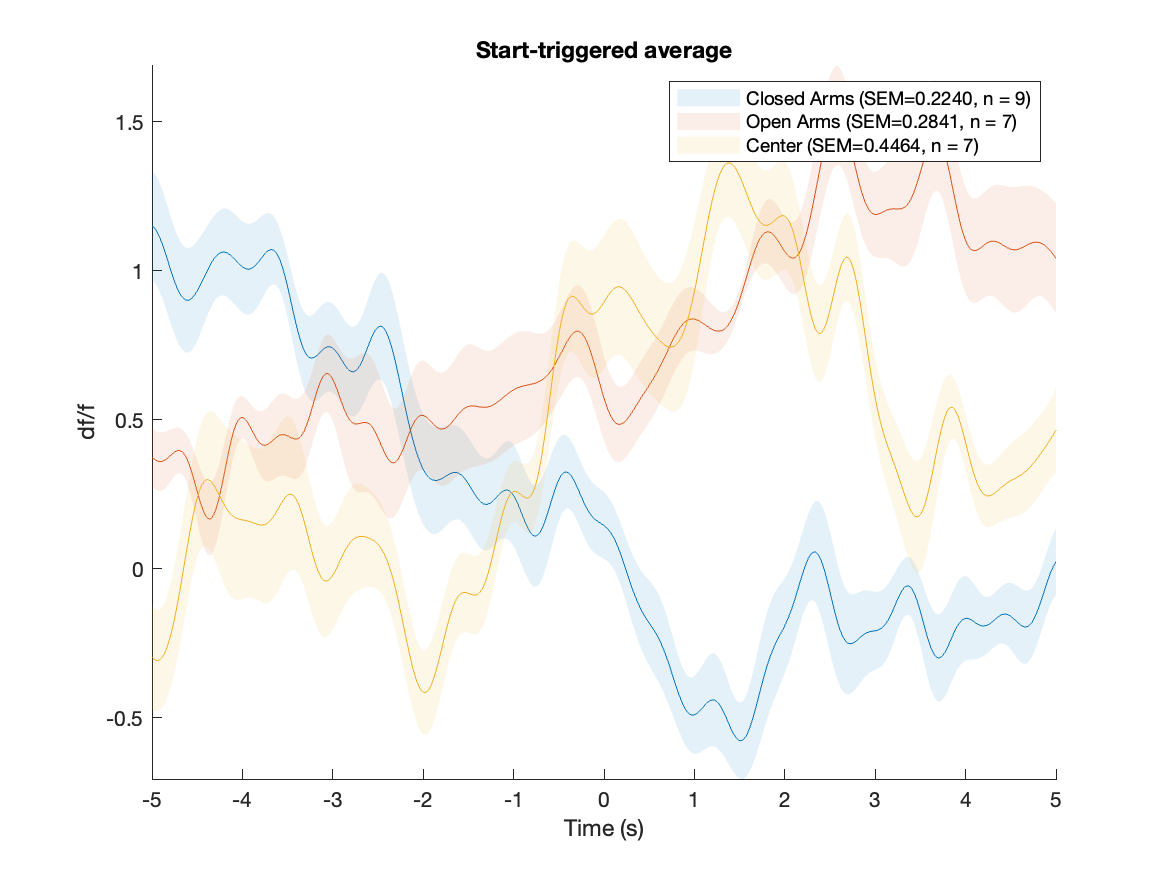

Supplement: Supplementary file 4 — Supplementary Software [file 41467_2024_45288_MOESM4_ESM.zip › Fiber Photometry Software/Outputs - from Sample Data/EPM outputs/FPA starttriggered average.png]

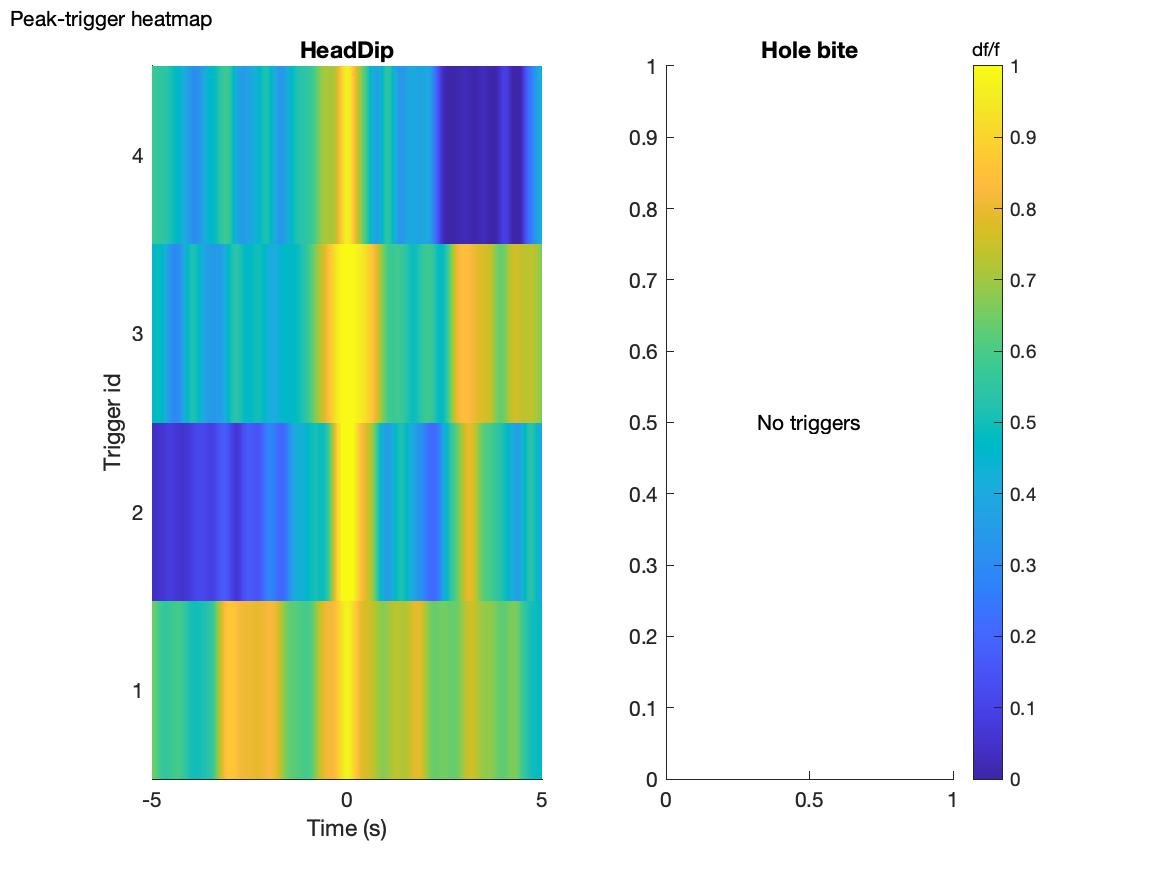

Supplement: Supplementary file 4 — Supplementary Software [file 41467_2024_45288_MOESM4_ESM.zip › Fiber Photometry Software/Outputs - from Sample Data/Holeboard outputs/HoleBoard_BORIS/Peaktrigger heatmap.png]

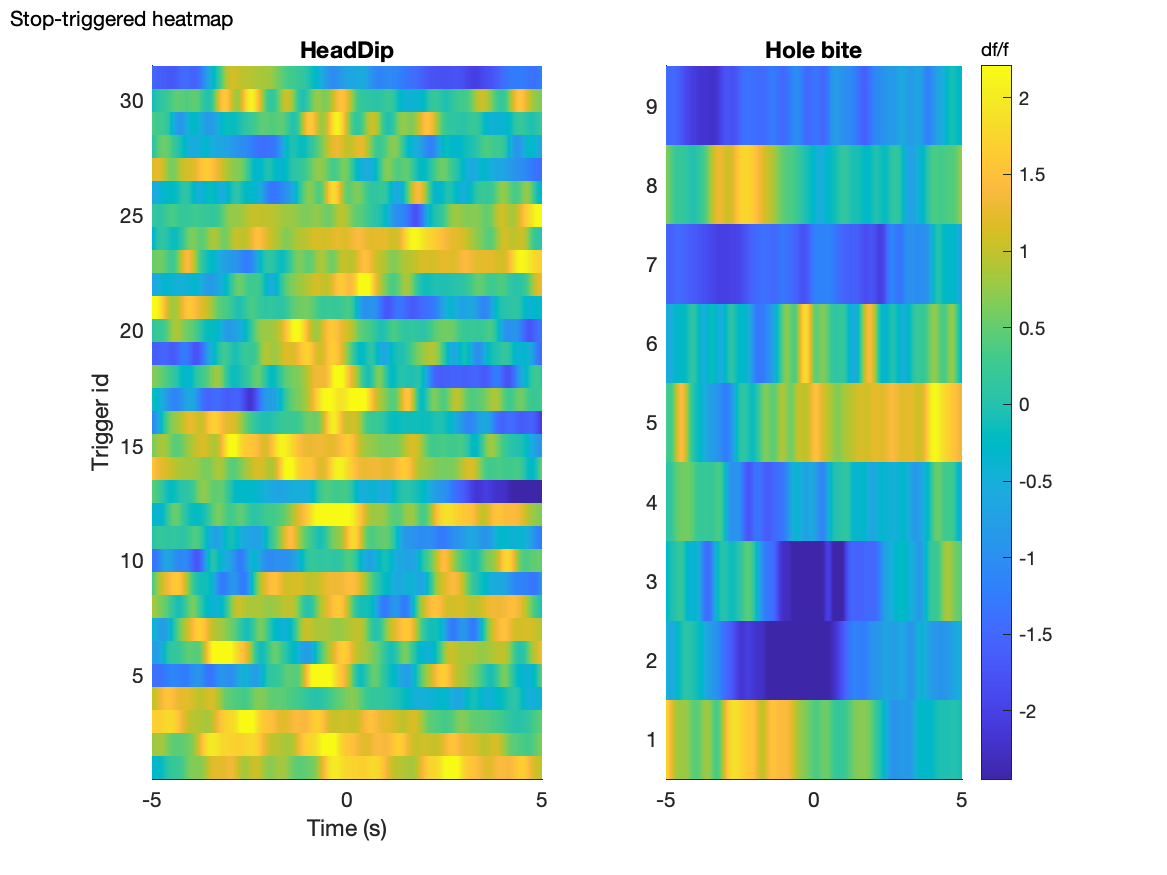

Supplement: Supplementary file 4 — Supplementary Software [file 41467_2024_45288_MOESM4_ESM.zip › Fiber Photometry Software/Outputs - from Sample Data/Holeboard outputs/HoleBoard_BORIS/Stoptriggered heatmap.png]

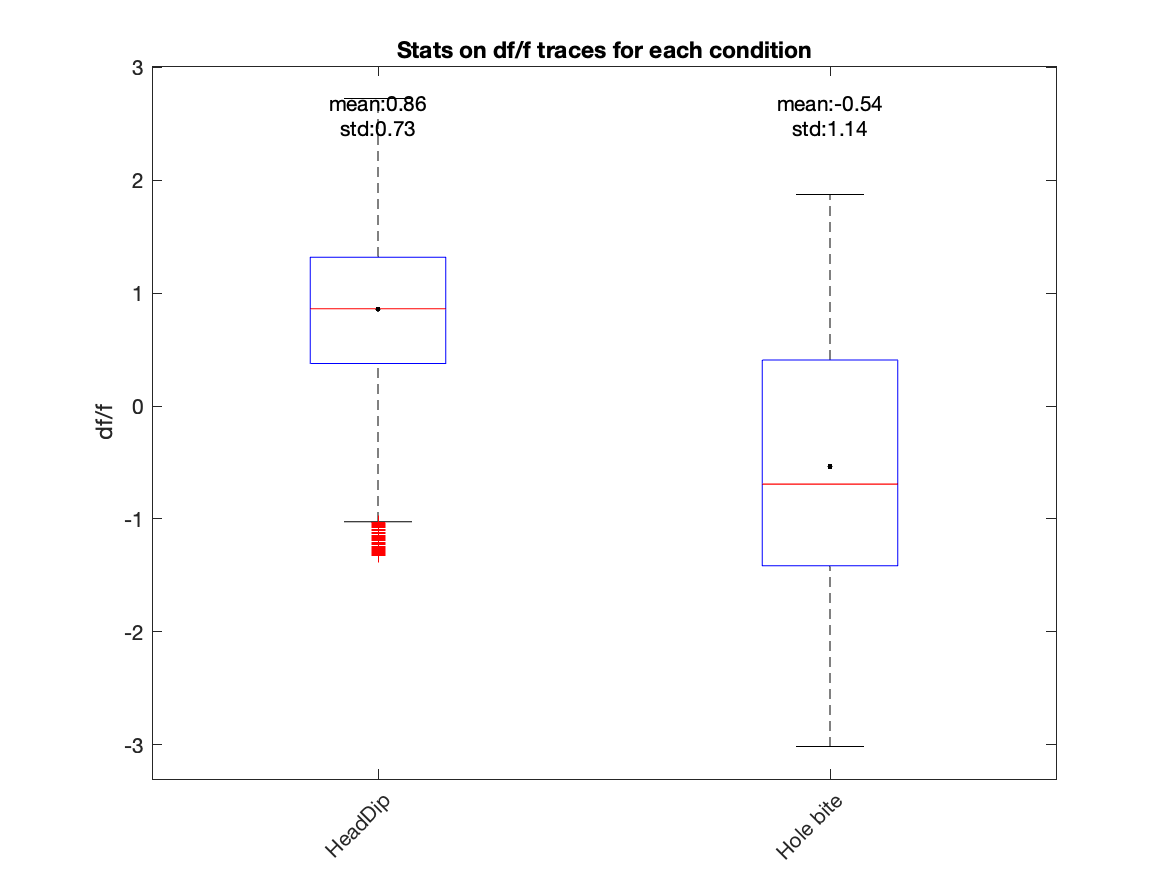

Supplement: Supplementary file 4 — Supplementary Software [file 41467_2024_45288_MOESM4_ESM.zip › Fiber Photometry Software/Outputs - from Sample Data/Holeboard outputs/HoleBoard_BORIS/FPA Boxplot.png]

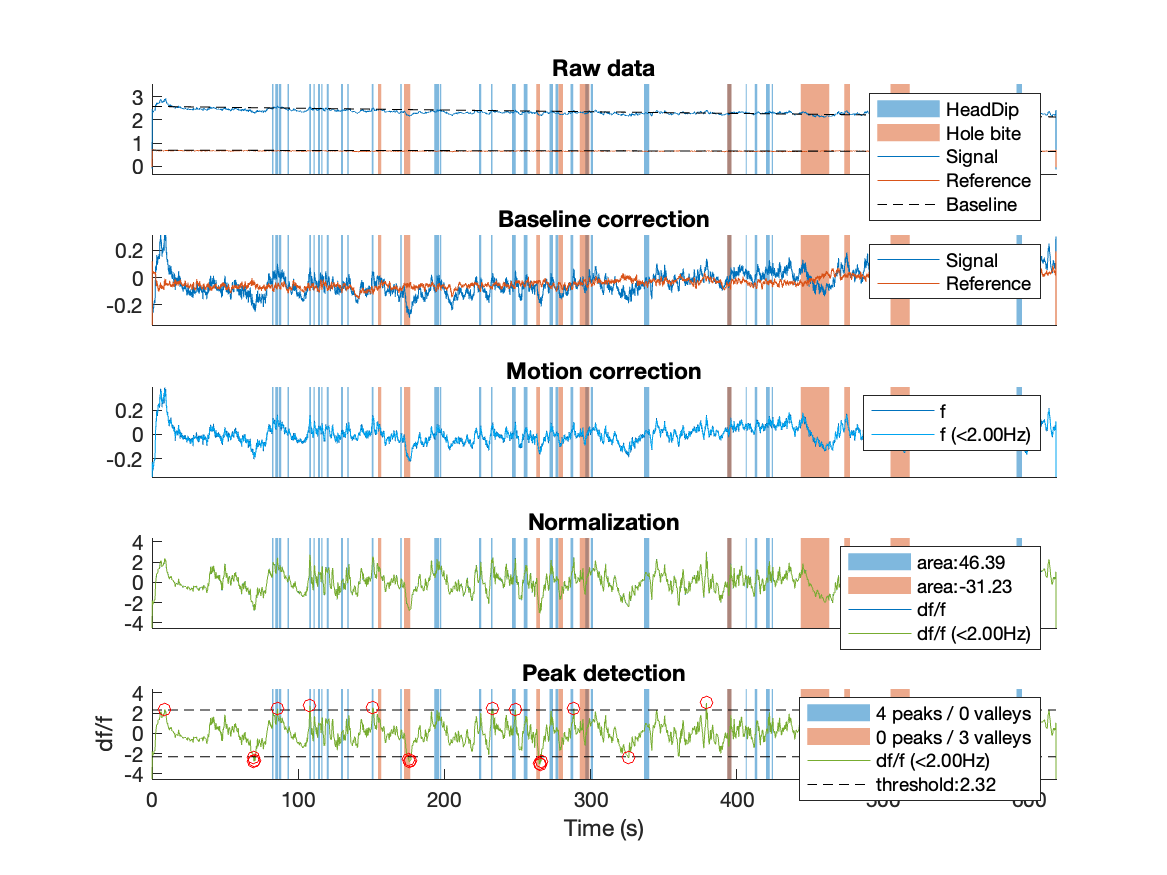

Supplement: Supplementary file 4 — Supplementary Software [file 41467_2024_45288_MOESM4_ESM.zip › Fiber Photometry Software/Outputs - from Sample Data/Holeboard outputs/HoleBoard_BORIS/FPA dff.png]

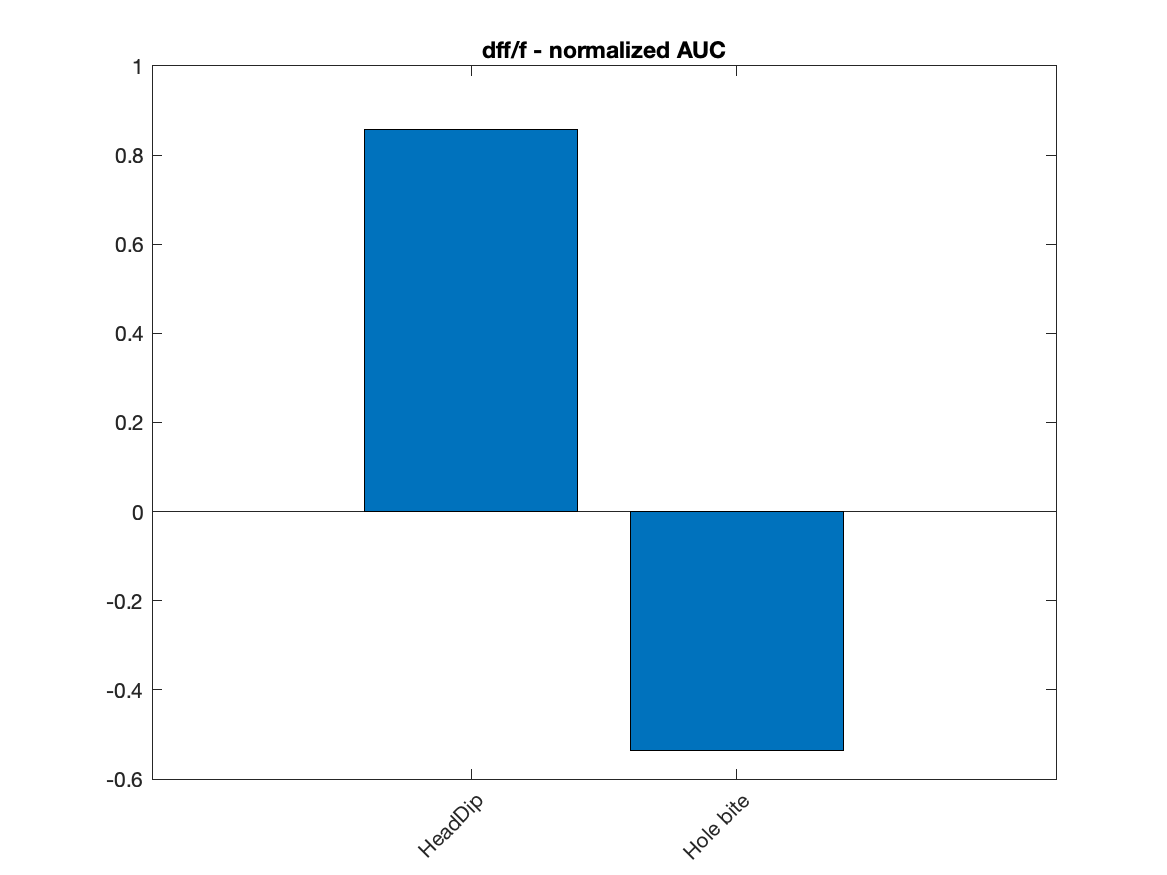

Supplement: Supplementary file 4 — Supplementary Software [file 41467_2024_45288_MOESM4_ESM.zip › Fiber Photometry Software/Outputs - from Sample Data/Holeboard outputs/HoleBoard_BORIS/FPA Normalized area under the curve.png]

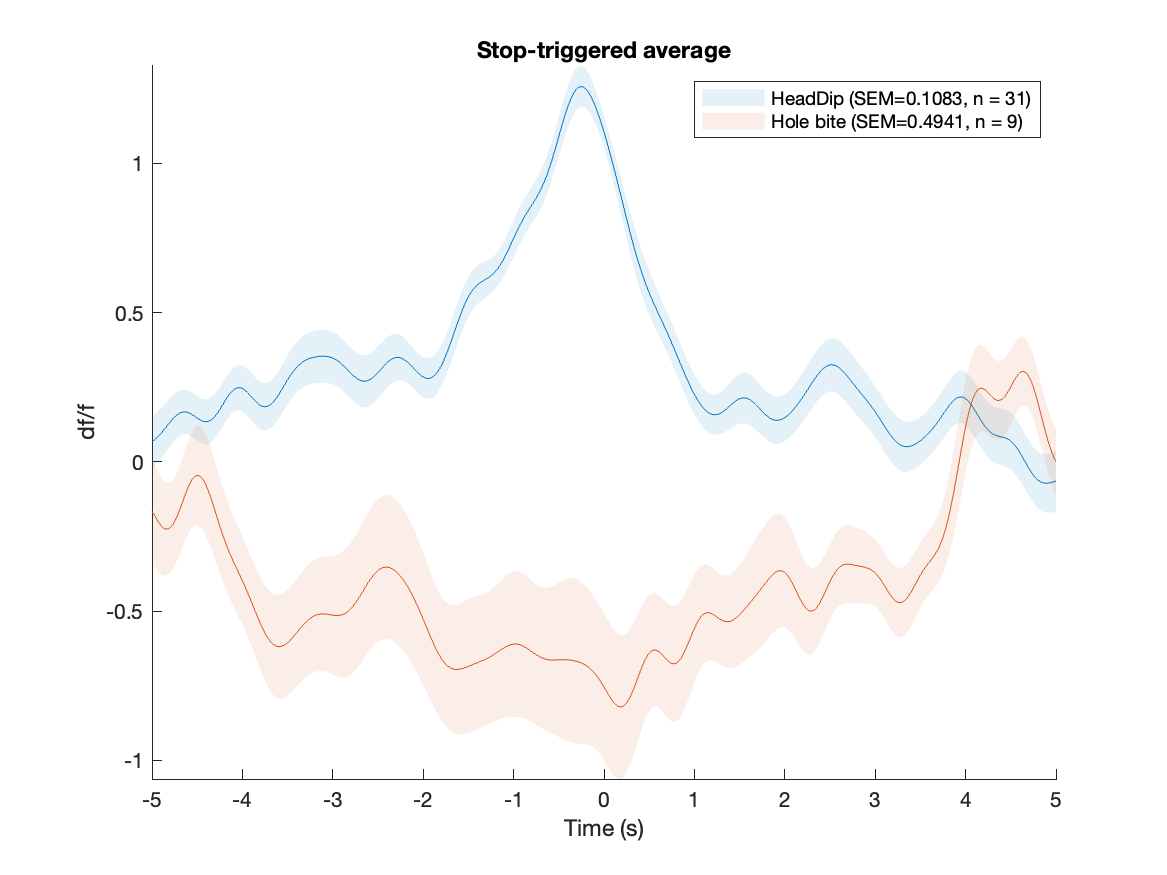

Supplement: Supplementary file 4 — Supplementary Software [file 41467_2024_45288_MOESM4_ESM.zip › Fiber Photometry Software/Outputs - from Sample Data/Holeboard outputs/HoleBoard_BORIS/FPA stoptriggered average.png]

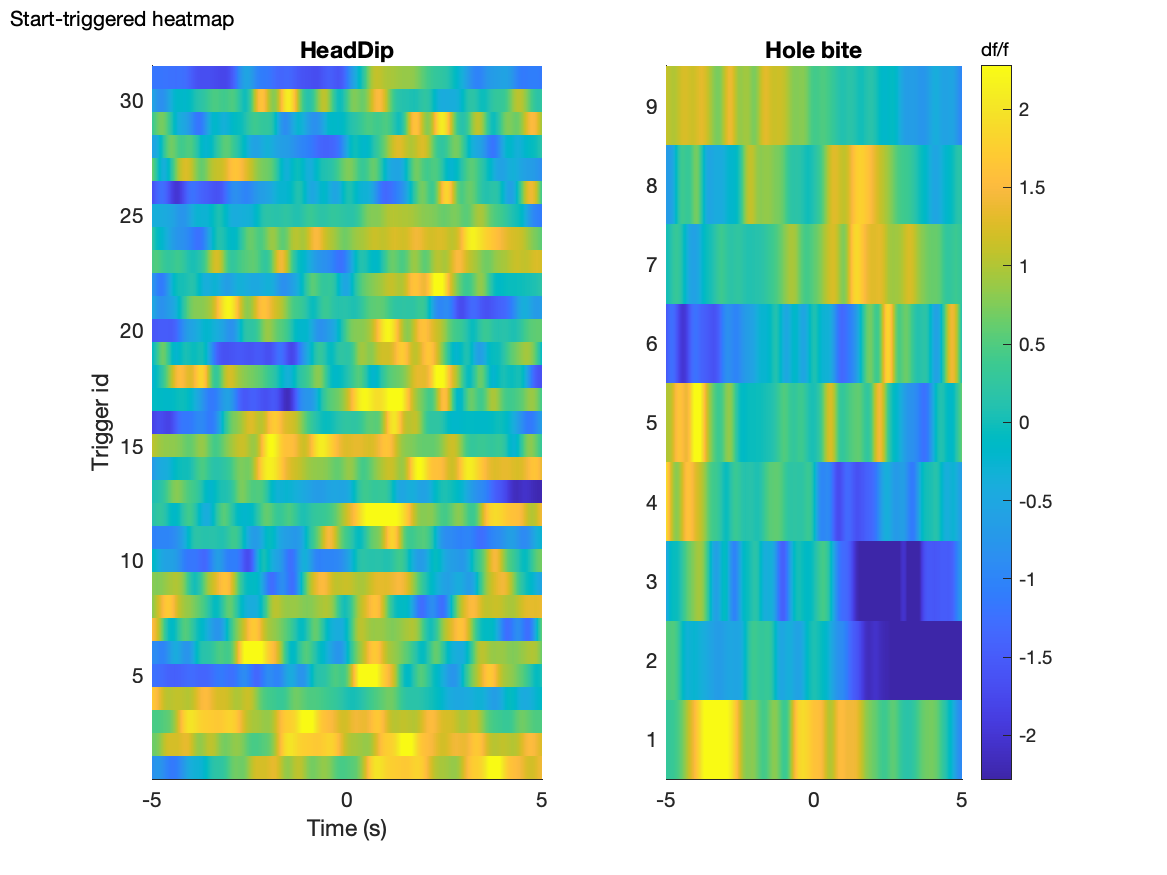

Supplement: Supplementary file 4 — Supplementary Software [file 41467_2024_45288_MOESM4_ESM.zip › Fiber Photometry Software/Outputs - from Sample Data/Holeboard outputs/HoleBoard_BORIS/Starttriggered heatmap.png]

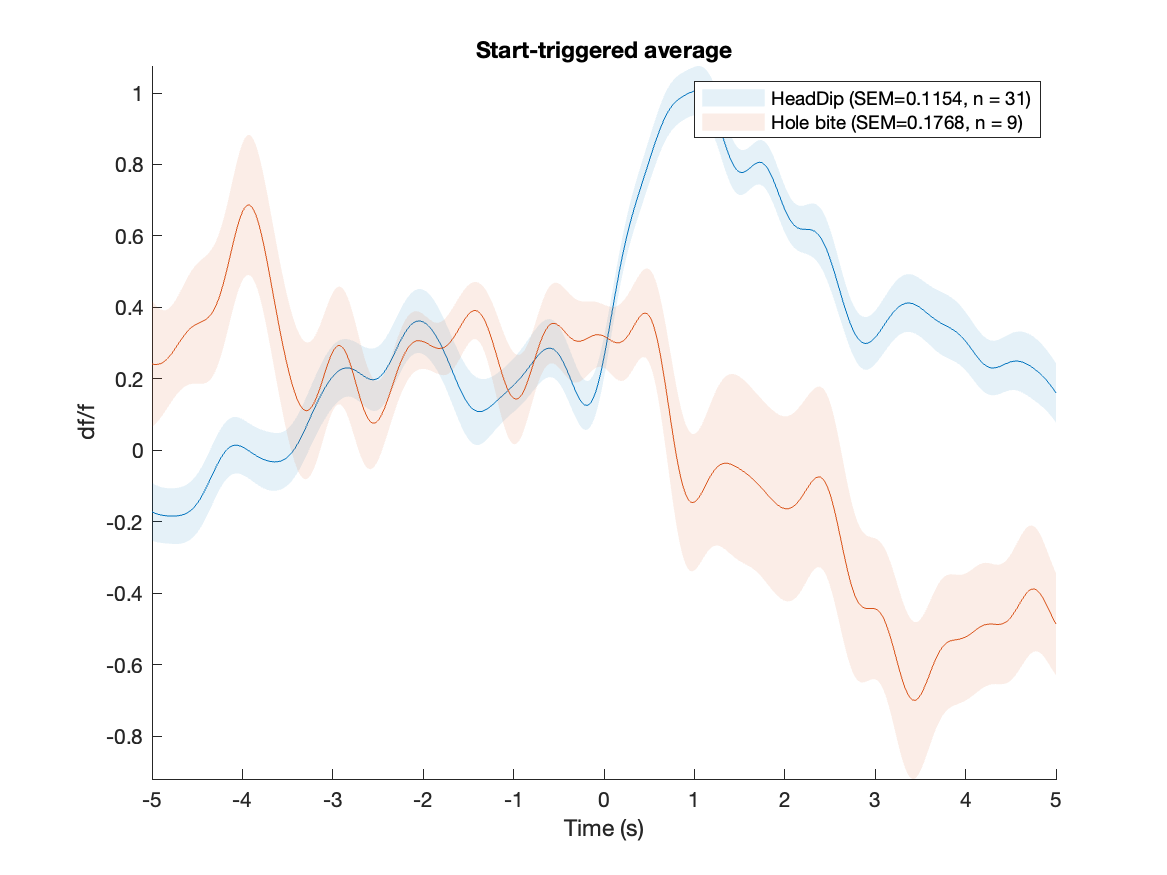

Supplement: Supplementary file 4 — Supplementary Software [file 41467_2024_45288_MOESM4_ESM.zip › Fiber Photometry Software/Outputs - from Sample Data/Holeboard outputs/HoleBoard_BORIS/FPA starttriggered average.png]

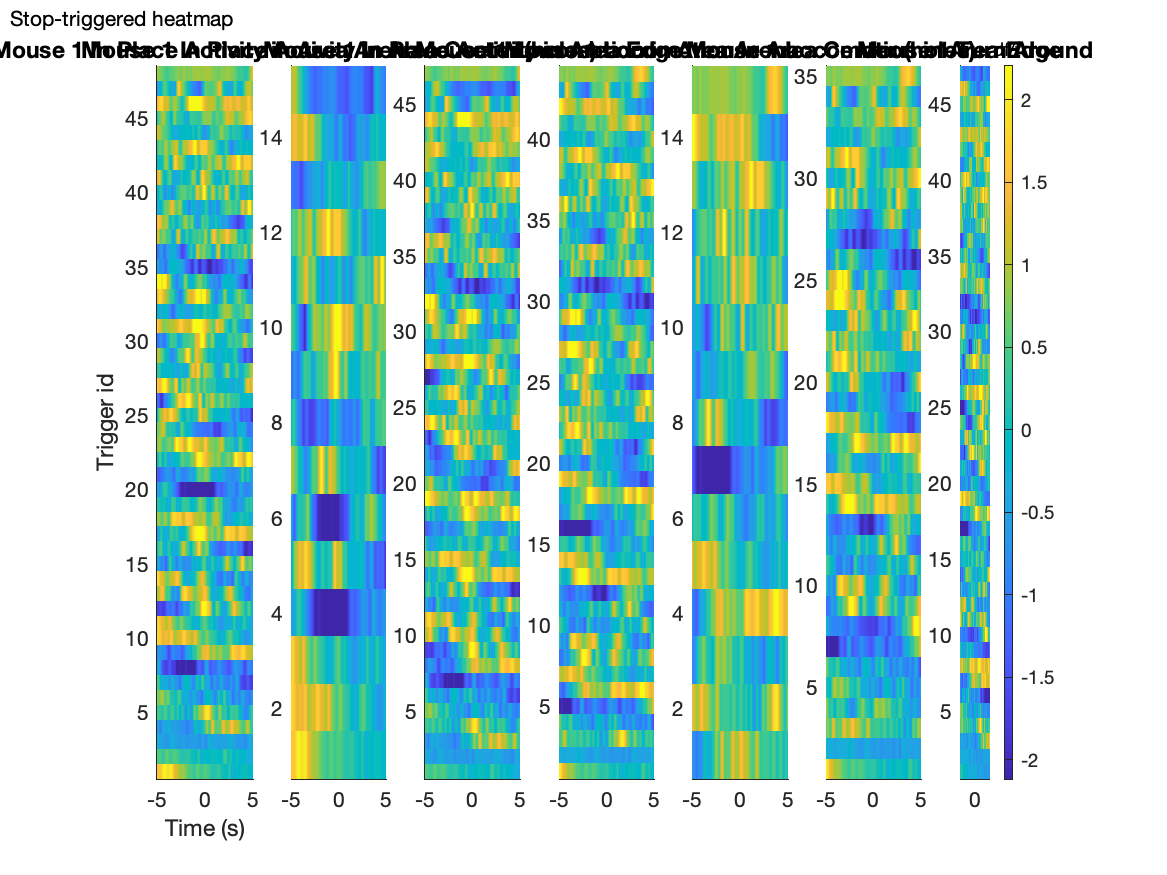

Supplement: Supplementary file 4 — Supplementary Software [file 41467_2024_45288_MOESM4_ESM.zip › Fiber Photometry Software/Outputs - from Sample Data/Holeboard outputs/Holeboard_cleversys/Stoptriggered heatmap.png]

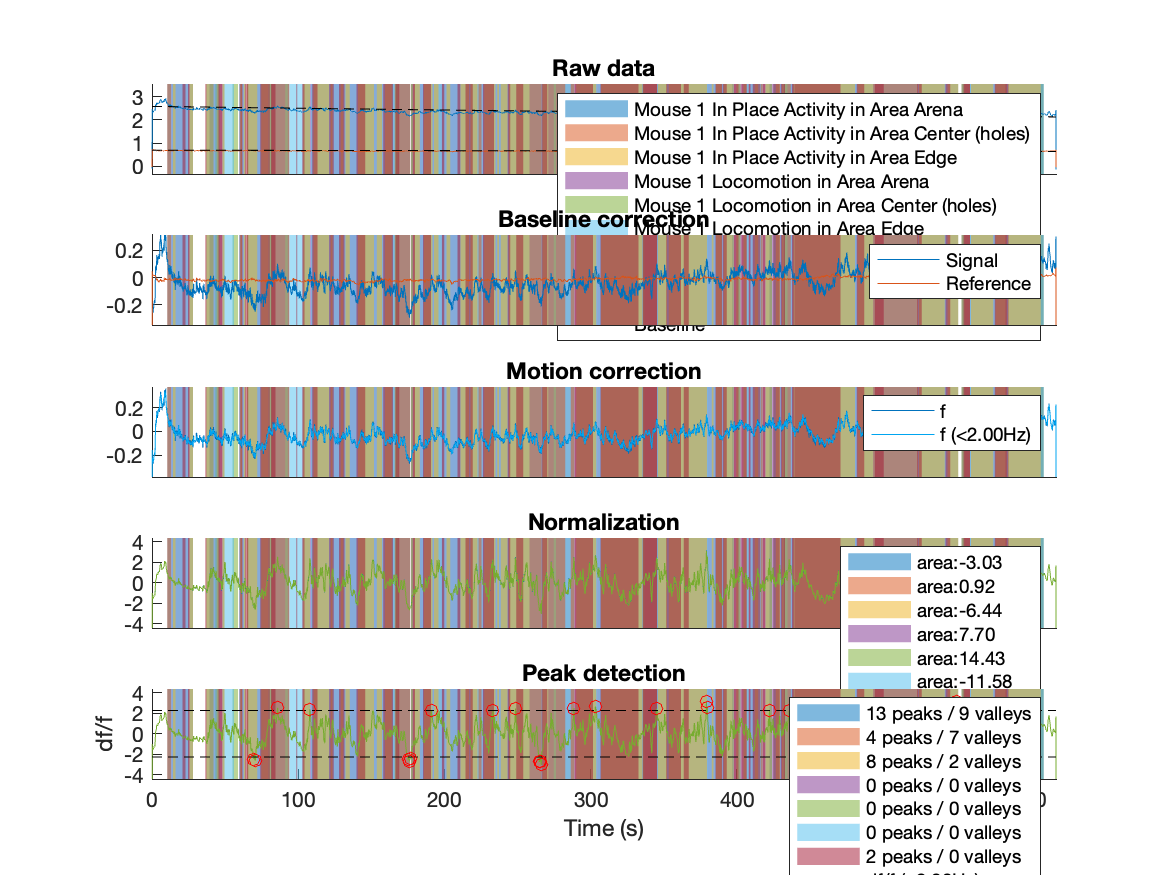

Supplement: Supplementary file 4 — Supplementary Software [file 41467_2024_45288_MOESM4_ESM.zip › Fiber Photometry Software/Outputs - from Sample Data/Holeboard outputs/Holeboard_cleversys/FPA dff.png]

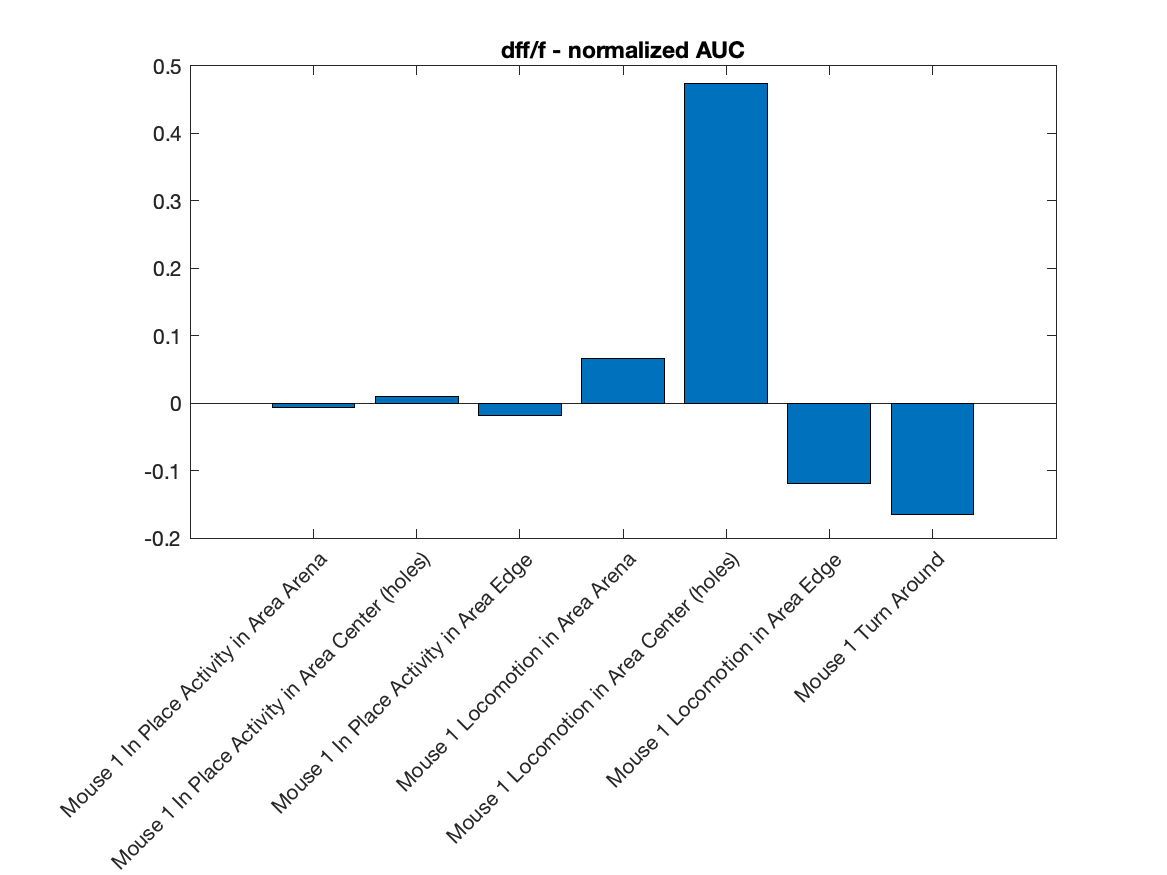

Supplement: Supplementary file 4 — Supplementary Software [file 41467_2024_45288_MOESM4_ESM.zip › Fiber Photometry Software/Outputs - from Sample Data/Holeboard outputs/Holeboard_cleversys/FPA Normalized area under the curve.png]

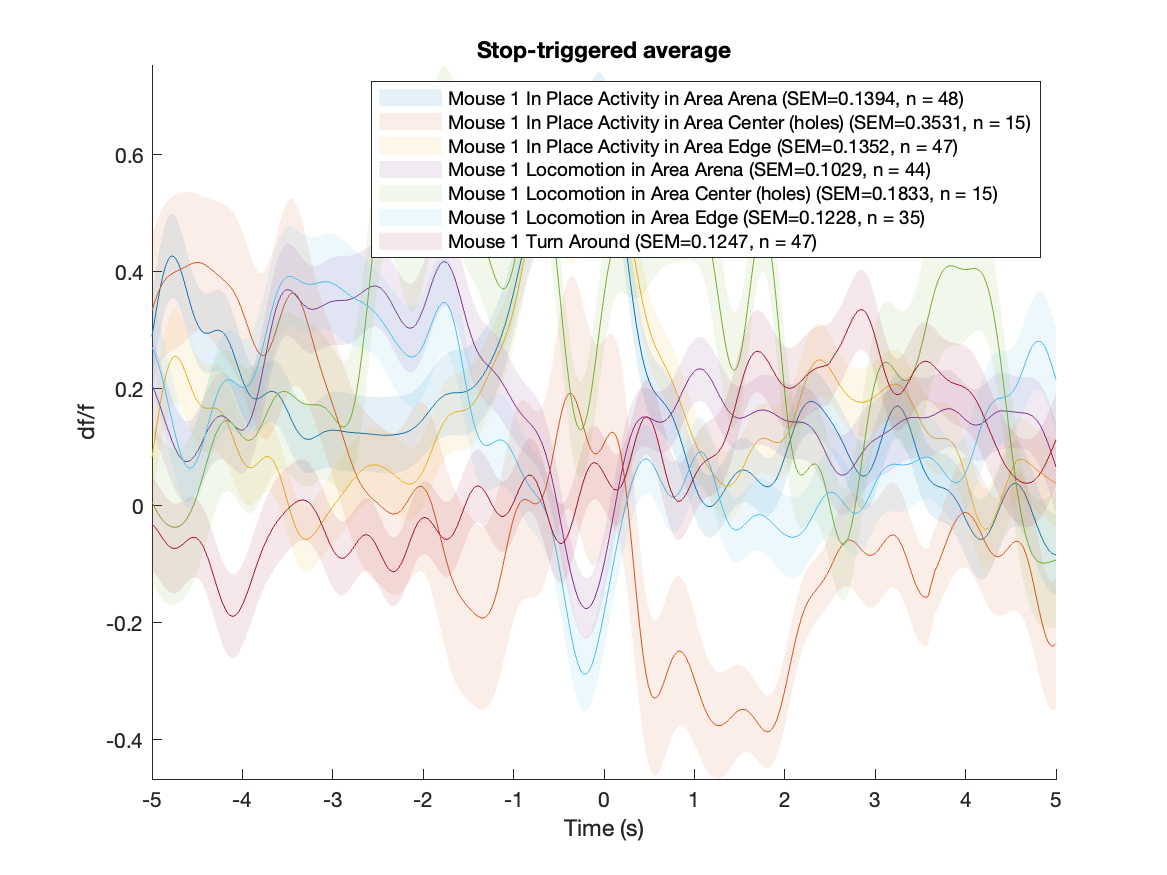

Supplement: Supplementary file 4 — Supplementary Software [file 41467_2024_45288_MOESM4_ESM.zip › Fiber Photometry Software/Outputs - from Sample Data/Holeboard outputs/Holeboard_cleversys/FPA stoptriggered average.png]

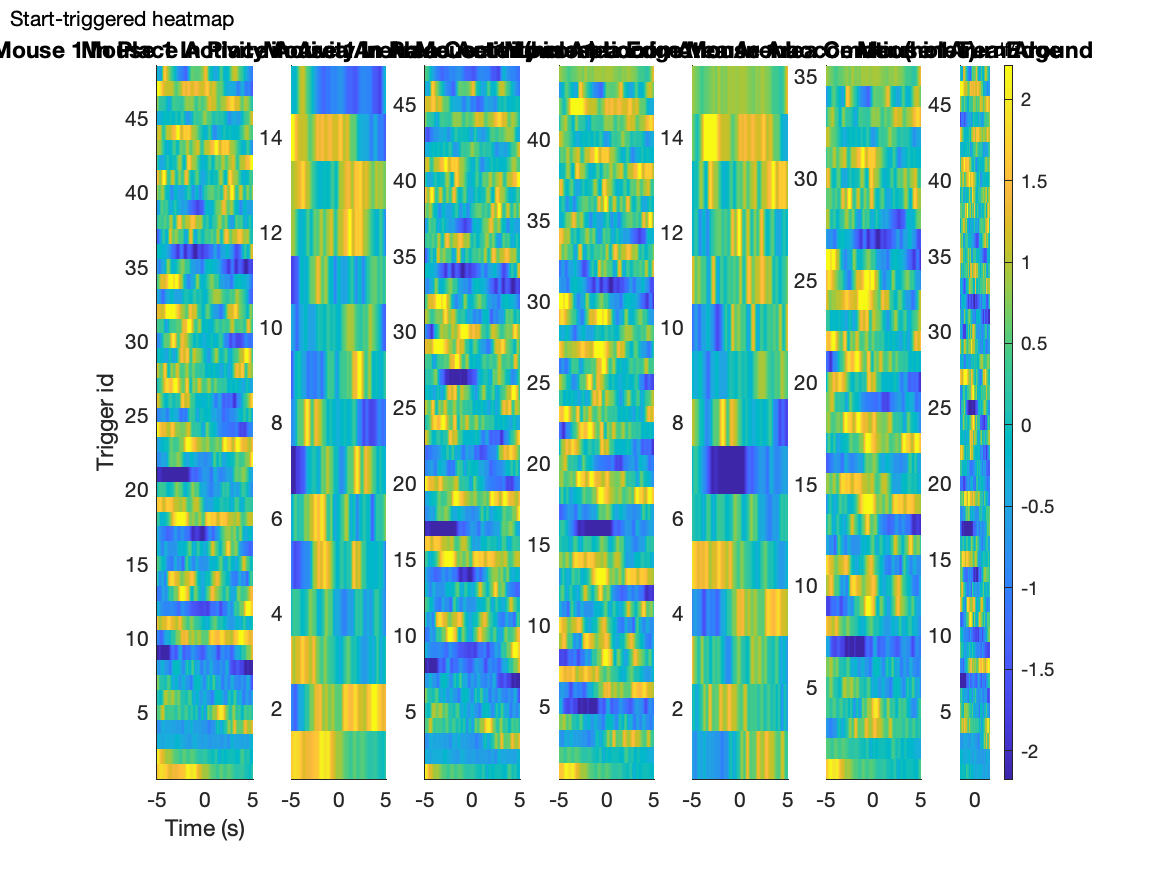

Supplement: Supplementary file 4 — Supplementary Software [file 41467_2024_45288_MOESM4_ESM.zip › Fiber Photometry Software/Outputs - from Sample Data/Holeboard outputs/Holeboard_cleversys/Starttriggered heatmap.png]

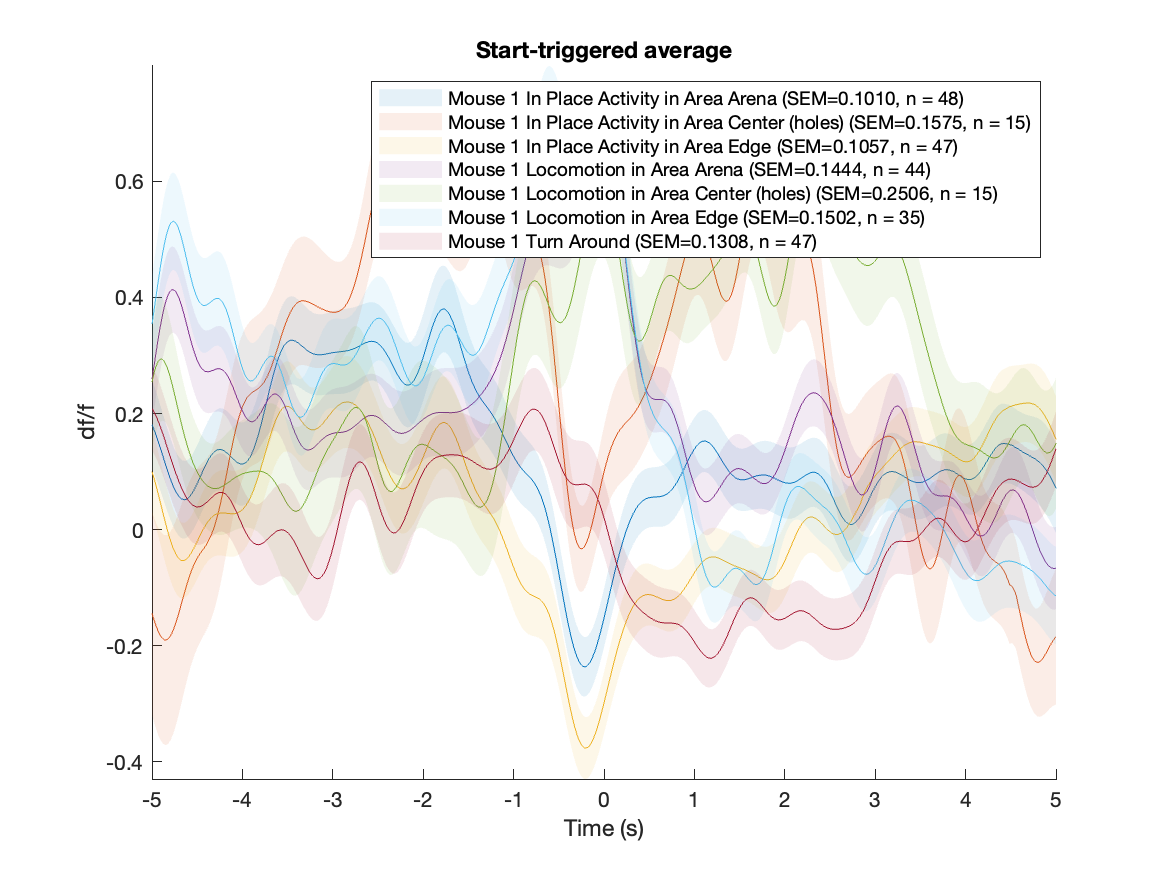

Supplement: Supplementary file 4 — Supplementary Software [file 41467_2024_45288_MOESM4_ESM.zip › Fiber Photometry Software/Outputs - from Sample Data/Holeboard outputs/Holeboard_cleversys/FPA starttriggered average.png]

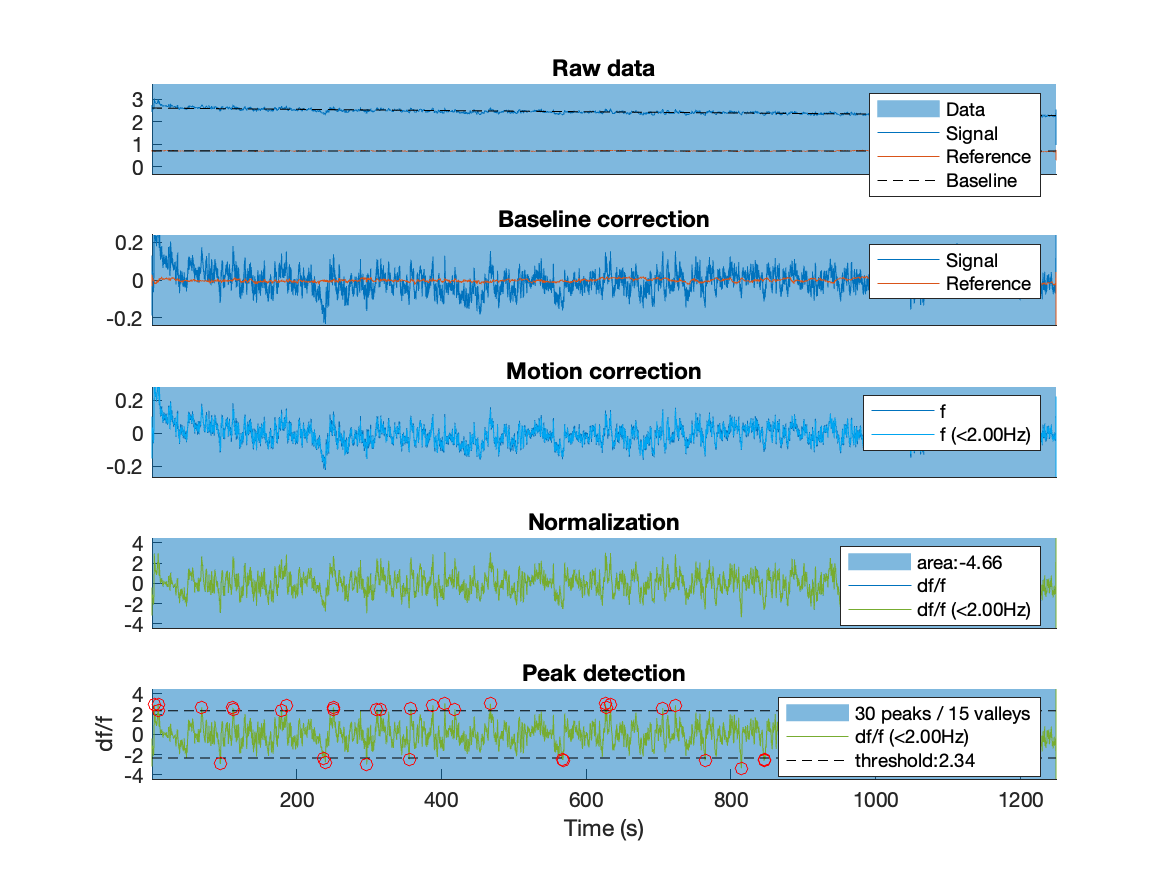

Supplement: Supplementary file 4 — Supplementary Software [file 41467_2024_45288_MOESM4_ESM.zip › Fiber Photometry Software/Outputs - from Sample Data/OFT outputs/mZ1FP1-1L_2_OFT - exported/FPA dff.png]

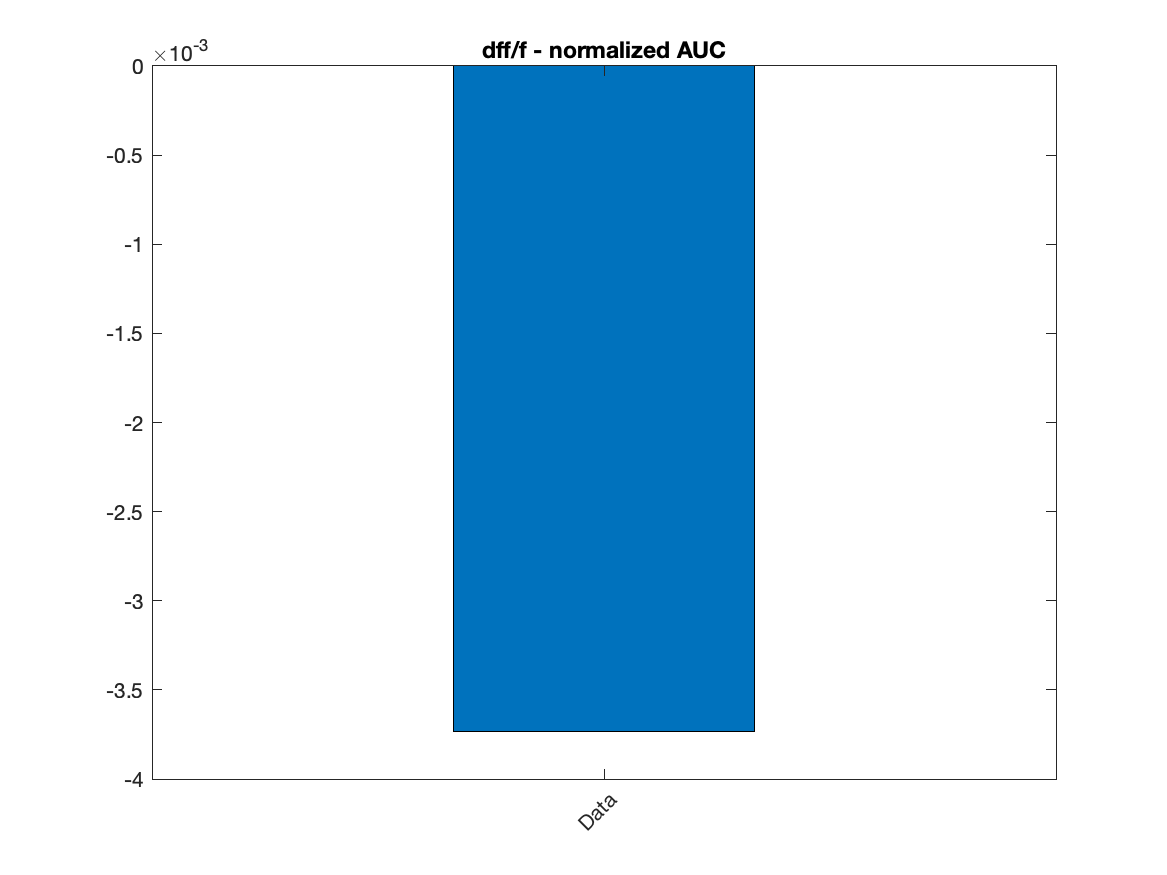

Supplement: Supplementary file 4 — Supplementary Software [file 41467_2024_45288_MOESM4_ESM.zip › Fiber Photometry Software/Outputs - from Sample Data/OFT outputs/mZ1FP1-1L_2_OFT - exported/FPA Normalized area under the curve.png]
