# Supplementary material for: Inhibitory medial zona incerta pathway drives exploratory behavior by inhibiting glutamatergic cuneiform neurons
Source: Nat Commun. 2024 Feb 7;15:1160. doi: 10.1038/s41467-024-45288-x (PMC10850156; doi:10.1038/s41467-024-45288-x)
Supplement: Supplementary file 5 — Reporting Summary [file 41467_2024_45288_MOESM5_ESM.pdf]

nature portfolio

Corresponding author(s): Dr. Patrick Whelan

Last updated by author(s): December 22, 2023

Reporting Summary

Nature Portfolio wishes to improve the reproducibility of the work that we publish. This form provides structure for consistency and transparency in reporting. For further information on Nature Portfolio policies, see our Editorial Policies and the Editorial Policy Checklist.

Statistics

For all statistical analyses, confirm that the following items are present in the figure legend, table legend, main text, or Methods section.

n/a	Confirmed	
☐	☒	The exact sample size (*n*) for each experimental group/condition, given as a discrete number and unit of measurement
☐	☒	A statement on whether measurements were taken from distinct samples or whether the same sample was measured repeatedly
☐	☒	The statistical test(s) used AND whether they are one- or two-sided<br>*Only common tests should be described solely by name; describe more complex techniques in the Methods section.*
☒	☐	A description of all covariates tested
☐	☒	A description of any assumptions or corrections, such as tests of normality and adjustment for multiple comparisons
☐	☒	A full description of the statistical parameters including central tendency (e.g. means) or other basic estimates (e.g. regression coefficient) AND variation (e.g. standard deviation) or associated estimates of uncertainty (e.g. confidence intervals)
☐	☒	For null hypothesis testing, the test statistic (e.g. F, t, r) with confidence intervals, effect sizes, degrees of freedom and P value noted<br>*Give P values as exact values whenever suitable.*
☒	☐	For Bayesian analysis, information on the choice of priors and Markov chain Monte Carlo settings
☒	☐	For hierarchical and complex designs, identification of the appropriate level for tests and full reporting of outcomes
☒	☐	Estimates of effect sizes (e.g. Cohen's *d*, Pearson's *r*), indicating how they were calculated

Our web collection on statistics for biologists contains articles on many of the points above.

Software and code

Policy information about availability of computer code

Data collection	Topscan version 3.0 from Clever-sys for behavioral data<br>Doric neuroscience studio (V5) software for fiber photometry<br>Spike Gadgets (MCU) for cell recording
Data analysis	MATLAB R2022<br>BORIS version 8.2<br>FIJI version 1.53f51<br>Illastik version 1.3.3 post3<br>QuPath version 0.2.3<br>Prism version 9

For manuscripts utilizing custom algorithms or software that are central to the research but not yet described in published literature, software must be made available to editors and reviewers. We strongly encourage code deposition in a community repository (e.g. GitHub). See the Nature Portfolio guidelines for submitting code & software for further information.

Data

Policy information about availability of data

All manuscripts must include a data availability statement. This statement should provide the following information, where applicable:

- Accession codes, unique identifiers, or web links for publicly available datasets
- A description of any restrictions on data availability
- For clinical datasets or third party data, please ensure that the statement adheres to our policy

Source data pertaining to the manuscript are available on Open Science Framework (OSF). https://doi.org/10.17605/OSF.IO/AU7ZE
Databases: Allen Reference Atlas (https://doi.org/10.1111/j.1601-183X.2009.00552.x)

Research involving human participants, their data, or biological material

Reporting on sex and gender	na
Reporting on race, ethnicity, or other socially relevant groupings	na
Population characteristics	na
Recruitment	na
Ethics oversight	na

Note that full information on the approval of the study protocol must also be provided in the manuscript.

Field-specific reporting

Please select the one below that is the best fit for your research. If you are not sure, read the appropriate sections before making your selection.

☒ Life sciences ☐ Behavioural & social sciences ☐ Ecological, evolutionary & environmental sciences

For a reference copy of the document with all sections, see nature.com/documents/nr-reporting-summary-flat.pdf

Life sciences study design

All studies must disclose on these points even when the disclosure is negative.

Sample size	No sample size calculation was performed. The sample size were similar to those reported in previous publications, PubMed 30364230, Pubmed 25343491 and Pubmed 29789702
Data exclusions	MIce that failed to meet crieria for viral injection or optic fiber placement were excluded from further analysis.
Replication	Data was collected from more than one batch of mice and experiments were replicated. Experiments and data analysis were done by different experimenters blindly. All the histological experiments and images were collected from at least 3 mice, except for 2 mice in fig 3a retrograde tracing of GABAergic afferents from mZI to the CnF in Vgat-IRES-Cre mouse line.
Randomization	All animal were randomly assigned to different groups
Blinding	The investigators were blinded to group allocation during data collection and analysis. The data was analyzed blindly if group allocation was clear during optogenetic experiments.

Reporting for specific materials, systems and methods

We require information from authors about some types of materials, experimental systems and methods used in many studies. Here, indicate whether each material, system or method listed is relevant to your study. If you are not sure if a list item applies to your research, read the appropriate section before selecting a response.

Materials & experimental systems

n/a	Involved in the study
☐	☒ Antibodies
☒	☐ Eukaryotic cell lines
☒	☐ Palaeontology and archaeology
☐	☒ Animals and other organisms
☒	☐ Clinical data
☒	☐ Dual use research of concern
☒	☐ Plants

Methods

n/a	Involved in the study
☒	☐ ChIP-seq
☒	☐ Flow cytometry
☒	☐ MRI-based neuroimaging

Antibodies

Antibodies used

Chicken anti-Green Fluorescent Protein Aves labs (#GFP-1010) (Lot GFP3717982, dilution 1:3000)
Goat anti-Choline Acetyltransferase Millipore Sigma (#AB144P) (Lot 3789368, dilution 1:500)
Alexa Fluor® 488 AffiniPure Donkey Anti-Chicken IgY (IgG) (H+L) Jackson ImmunoResearch (#703-545-155), (Lot 154923, dilution 1:1000
Alexa Fluor® 647 Donkey anti-Goat IgG (H+L) Cross-Adsorbed Secondary Antibody ThermoFisher Scientific (#A-21447), (Lot 2273780 dilution 1:1000

Validation

Chicken anti-Green Fluorescent Protein (Aves Labs Cat# GFP-1010, RRID:AB_2307313)
Goat anti-Choline Acetyltransferase (Millipore Cat# AB144P, RRID:AB_2079751)
Alexa Fluor® 488 AffiniPure Donkey Anti-Chicken IgY (IgG) (H+L) (Jackson ImmunoResearch Labs Cat# 703-545-155, RRID:AB_2340375)
Alexa Fluor® 647 Donkey anti-Goat IgG (H+L) Cross-Adsorbed Secondary Antibody (Thermo Fisher Scientific Cat# A-21447, RRID:AB_2535864)

Animals and other research organisms

Policy information about studies involving animals; ARRIVE guidelines recommended for reporting animal research, and Sex and Gender in Research

Laboratory animals

Adult male mice aged between 8-16 weeks were used. Mice were housed on a 12:12 hour light:dark schedule (lights on 07:00 – off 19:00) with ad libitum access to food and water. Relative Humidity 29%, Temperature 20 C.: Strains: B6J.129S6(FVB)-Slc17a6 & Slc32a1

Wild animals

No wild mice were used

Reporting on sex

Only male mice were used. Previous work has shown no differences between male and female mice in the MLR.

Field-collected samples

No field-collected samples were used in this study

Ethics oversight

All animal experiments were approved by the University of Calgary Health Sciences Animal Care Committee (Protocol: AC19-0035) and were conducted in accordance with the guidelines of the Canadian Council for Animal Care.

Note that full information on the approval of the study protocol must also be provided in the manuscript.

Plants

Seed stocks

na

Novel plant genotypes

na

Authentication

na
